# An agricultural triazole induces genomic instability and haploid cell formation in the human fungal pathogen *Candida tropicalis*

**DOI:** 10.1371/journal.pbio.3003062

**Published:** 2025-04-01

**Authors:** Tianren Hu, Qiushi Zheng, Chengjun Cao, Shuaihu Li, Yanfeng Huang, Zhangyue Guan, Lingyu Ji, Jian Bing, Han Du, Austin M. Perry, Clarissa J. Nobile, Bing Li, Haiqing Chu, Guanghua Huang

**Affiliations:** 1 Department of Respiratory and Critical Care Medicine, Shanghai Pulmonary Hospital, School of Medicine, Tongji University, Shanghai, China; 2 Shanghai Key Laboratory of Tuberculosis, Shanghai Pulmonary Hospital, School of Medicine, Tongji University, Shanghai, China; 3 Department of infectious diseases, Huashan Hospital, Shanghai Institute of Infectious Disease and Biosecurity and State Key Laboratory of Genetic Engineering, School of Life Sciences, Fudan University, Shanghai, China; 4 College of Pharmaceutical Sciences, Southwest University, Chongqing, China; 5 Institutes of Biomedical Sciences, Fudan University, Shanghai, China; 6 Department of Molecular and Cell Biology, University of California, Merced, California, United States of America; 7 Quantitative and Systems Biology Graduate Program, University of California, Merced, California, United States of America; 8 Health Sciences Research Institute, University of California, Merced, California, United States of America; Duke University Medical Center, UNITED STATES OF AMERICA

## Abstract

The human fungal pathogen *Candida tropicalis* is widely distributed in clinical and natural environments. It is known to be an obligate diploid organism with an incomplete and atypical sexual cycle. Azole-resistant *C. tropicalis* isolates have been observed with increasing prevalence in many countries in recent years. Here, we report that tebuconazole (TBZ), a triazole fungicide widely used in agriculture, can induce ploidy plasticity and the formation of haploid cells in *C. tropicalis*. The evolved *C. tropicalis* strains with ploidy variations exhibit a cross-resistance between TBZ and standard azoles used in clinical settings (such as fluconazole and voriconazole). Similar to its diploid cells, these newly discovered *C. tropicalis* haploid cells are capable of undergoing filamentation, white-opaque switching, and mating. However, compared to its diploid cells, these haploid *C. tropicalis* cells grow more slowly under in vitro culture conditions and are less virulent in a mouse model of systemic infection. Interestingly, flow cytometry analysis of a clinical strain with extremely low genome heterozygosity indicates the existence of natural *C. tropicalis* haploids. Discovery of this *C. tropicalis* haploid state sheds new light into the biology and genetic plasticity of *C. tropicalis* and could provide the framework for the development of new genetic tools in the field.

## Abstract 2

Candida tropicalis is a important human pathogen that is increasingly resistant to azole antifungals. Although C. tropicalis was thought to be strictly diploid, this study shows that a widely used agricultural fungicide induces ploidy plasticity, leading to the formation of haploid cells that are cross-resistant to antifungal drugs.

## Introduction

The opportunistic human fungal pathogen *Candida tropicalis* causes both superficial and life-threatening invasive infections, and represents the second or third etiological agent of candidemia depending on the patient population [1–4]. Recent studies have reported increases in incidences of *C. tropicalis* infections as well as azole resistance properties in *C. tropicalis* isolates collected from hospitals throughout China and Japan [2,3,5,6]. For example, between 2009 and 2018, mainland China experienced a dramatic increase in the resistance rates of *C. tropicalis* infections to fluconazole (from 5.7% to 31.8%) and to voriconazole (from 5.7% to 29.1%) [3]. The reasons for these notable increases in *C. tropicalis* azole resistance remain unclear. As a result, *C. tropicalis* has been gaining considerable attention, especially in Asia-Pacific countries and Latin America [5,6].

Unlike its closely related species *Candida albicans*, which is predominantly associated with humans and other mammalian hosts as a commensal or as an opportunistic pathogen, *C*. *tropicalis* is omnipresent in a wide variety of niches, including soil, beach sand, fruits, plants, in addition to being associated with humans and other mammalian hosts [2,4,7–11]. This widespread ecological distribution reflects the remarkable adaptive plasticity of *C. tropicalis* to thrive in ubiquitous environments.

Azoles are a class of antifungal drugs that are commonly used in both agricultural and clinical contexts [12,13]. Azoles are 14-α-demethylase inhibitors, which target Cyp51 (or Erg11) of the ergosterol biosynthetic pathway in fungi [13–15]. Fluconazole is the most widely used antifungal drug for treating *Candida* infections worldwide. Structurally similar azoles have been used in both agricultural and clinical contexts worldwide [12,13]. The azole tebuconazole (TBZ) is a broad-spectrum agricultural fungicide used in the prevention and treatment of fungal diseases in common food crops, such as fruits, grains, and vegetables [12,13]. Due to the widespread use of this fungicide, TBZ can accumulate and be extensively found in the environment and in foods [13,16].

Given that human fungal pathogens such as *Aspergillus fumigatus*, *Cryptococcus neoformans*, and *C. tropicalis* are generally present in the natural environment, the agricultural application of azole fungicides could induce a cross-resistance in these pathogens to clinical antifungals, promoting the spread of resistance [17–19]. In this study, we set out to evaluate the effect of TBZ on the genomic stability and development of fluconazole resistance in *C. tropicalis*. We performed experimental evolution assays by treating environmental and clinical isolates of *C. tropicalis* with TBZ. We found that exposure to TBZ resulted in genomic alterations and the generation of aneuploidy and haploidy in *C. tropicalis*, which has long been considered an obligate diploid yeast. In addition, we observed that like diploid *C. tropicalis* cells, the haploid *C. tropicalis* cells that were generated after exposure to TBZ could undergo morphological transitions and were mating competent. Furthermore, the evolved TBZ-resistant strains exhibited cross-resistance between agricultural and clinical azoles.

## Results

### Tebuconazole induces cross-resistance with clinical azoles in *C. tropicalis*

**Fig 1:**
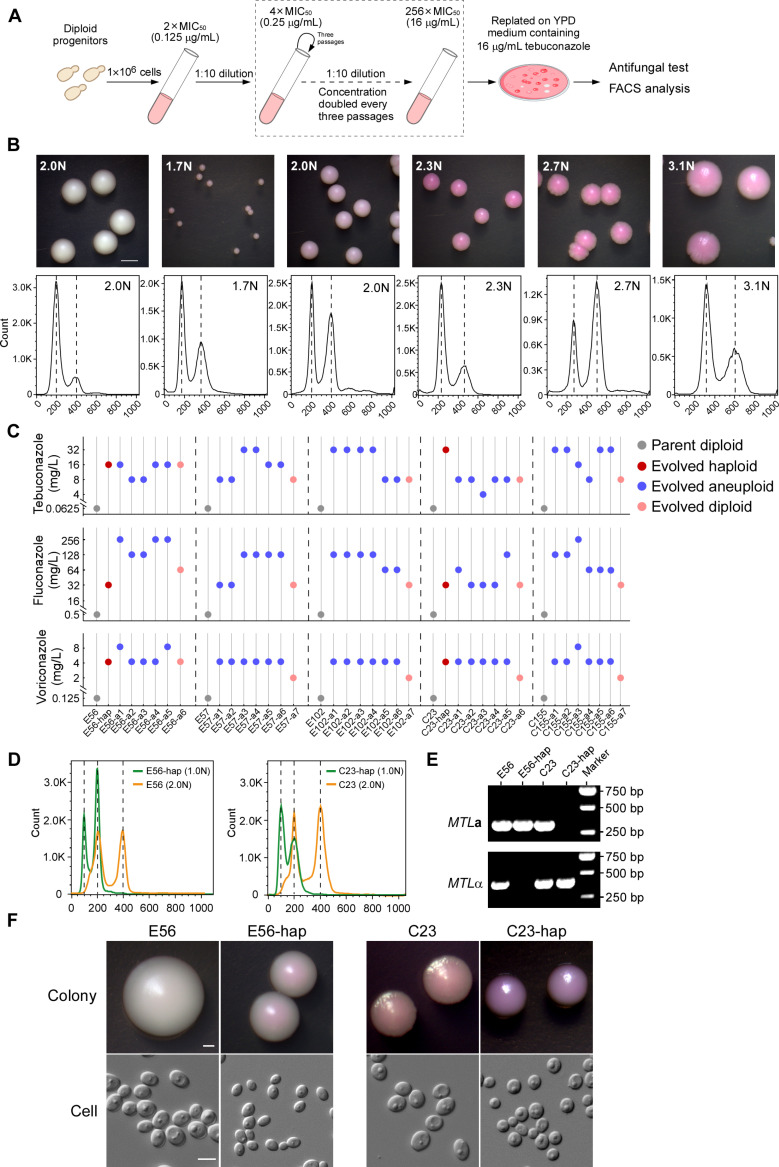
Tebuconazole induces ploidy variation and formation of haploid cells in *Candida tropicalis.* (**A**) Schematic diagram of in vitro induction of antifungal resistant strains with varied ploidy. A single colony of the *C. tropicalis* diploid progenitor was inoculated and cultured in RPMI-MOPS medium (containing 2% glucose) at 37 °C for 24 h. Then, 1 ×  10^6^ cells were inoculated into 5 mL fresh medium containing 0.125 μg/mL tebuconazole (2 ×  MIC_50_) and grown at 37 °C for 72 h. Subsequently, 0.5 mL of the culture (1:10 dilution) was transferred into 4.5 mL fresh medium with a doubled concentration of tebuconazole and incubated for 72 h. A total of three passages were performed under each concentration. After three passages at the same culture condition, the concentration of tebuconazole was doubled. The passages were repeated and the drug concentration was increased up to 16 μg/mL. Three independent replicates were performed. Fungal cells were finally harvested and replated onto YPD plates containing 16 μg/mL of tebuconazole at 37 °C for 4 days. Single colonies were then subject to further analysis. A control passage in the absence of tebuconazole was performed in parallel. (**B**) Colony morphology and flow cytometry analyses of representative evolved aneuploidy isolates. Fungal cells were grown on YPD medium containing 5 μg/mL phloxine B at 30 °C for 2 days. Scale bar, 2 mm. Flow cytometry analysis was performed to determine the DNA content of the strains. (**C**) Azole antifungal susceptibility of the evolved strains. In vitro susceptibility assays were performed according to the CLSI M27 (4th edition) protocols. The evolved isolates were derived from five independent diploid parental strains (E56, E57, E102, C23, and C155). (**D**) Flow cytometry analysis of the DNA content of the evolved *C. tropicalis* haploid and parental diploid strains. (**E**) PCR verification of the *MTL* configurations of the haploid and diploid strains. (**F**) Colony and cellular morphologies of the haploid and diploid strains. Cells were grown on YPD medium containing 5 μg/mL phloxine B at 30 °C for 2 days. Scale bars: 1 mm for colonies; 5 μm for cells. The data underlying this figure can be found in S3 Data. The flow cytometry files are available from the Figshare database (https://doi.org/10.6084/m9.figshare.28350296).

Since the fungicide TBZ is widely used in agriculture and could accumulate and persist in the environment, we set out to investigate whether this agrochemical could induce genomic variations in *C. tropicalis* that result in cross-resistance with clinical azoles. As shown in Fig 1A, we designed an experimental evolution assay to obtain azole-resistant cells with five initially TBZ-susceptible strains (E56, E57, E102, C23, and C155, TBZ MIC_50_ =  0.06 μg/mL). These strains were also initially susceptible to clinical azoles such as fluconazole (MIC_50_ =  0.5 μg/mL) and voriconazole (MIC_50_ =  0.125 μg/mL). The strains were treated with incrementally increasing concentrations of TBZ (from 0.125 to 16 μg/mL) in liquid medium and finally replated onto medium containing 16 μg/mL TBZ. In total, we selected 35 evolved *C. tropicalis* isolates from the TBZ-containing plates based on the colony morphology and growth state for further study (S1 Table). Antifungal susceptibility tests demonstrated that all evolved isolates were TBZ-resistant (MIC_50_ ≥  4.0 μg/mL). Of them, 11 isolates (31.4%) exhibited TBZ MIC_50_ values as high as 32 μg/mL. Interestingly, all the evolved strains exhibited increased resistance to commonly used clinical azoles, such as fluconazole (MIC_50_ of 32–256 μg/mL), voriconazole (MIC_50_ of 2–8 μg/mL), itraconazole (MIC_50_ of 1–2 μg/mL), and posaconazole (MIC_50_ of 1–4 μg/mL). However, no significant changes in susceptibility to caspofungin and amphotericin B were observed (S1 Data). These results suggest that TBZ treatment in *C. tropicalis* induces not only TBZ resistance but also cross-resistance to azole drugs (S1 Data). Moreover, we observed that the evolved drug-resistant strains exhibited a slower growth rate than the progenitor strain in the absence of antifungals, whereas they grew much better than the progenitor strain in the presence of antifungals (S1 Fig). Therefore, there could be a trade-off effect between cell growth and antifungal resistance in the evolved strains.

### Tebuconazole induces ploidy variation and the formation of haploid cells in *C. tropicalis*

**Fig 2:**
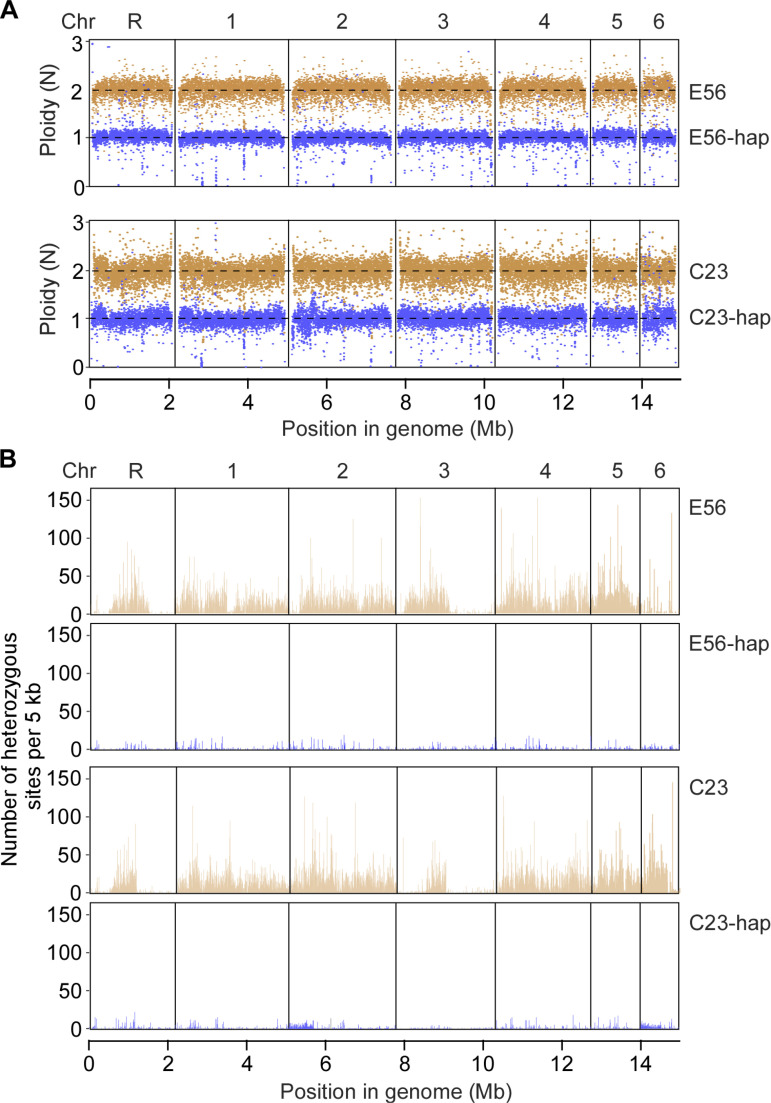
Ploidy and heterozygosity analyses of *Candida tropicalis* haploid and diploid strains. The ploidy states were initially examined using flow cytometry assays. (**A**) Scatter plot of the genome-wide copy number distribution. The *x*-axis represents the seven chromosomes and *y*-axis represents the relative level of copy number. Each point indicates an average copy number of a genomic segment of 1,000 bp across the genome based on the coverage analysis of the genomic data. (**B**) Relative heterozygosity levels of the haploid and diploid strains across the genome. *x*-axis, position in the genome. *y*-axis, number of heterozygous sites per 5 kb. Diploid strains, E56 and C23 (light brown); haploid strains, E56-hap and C23-hap (blue).

We observed that the colonies formed by the evolved *C. tropicalis* TBZ-resistant strains appeared pink or red on agar containing phloxine B, whereas their parental controls were white or light pink (Fig 1B). The cell sizes of the evolved strains also varied. Since both cell size and colony coloration are associated with karyotypes in pathogenic fungi [20,21], we next examined the genomic DNA content and performed whole genome sequencing analysis on all evolved strains. We found that the ploidy of these strains varied from 1.0 to 3.1 N (Fig 1 and S1 Table). Based on flow cytometry and whole genome sequencing analyses, we found that most of the evolved drug-resistant strains had an altered ploidy (Fig 1C). Approximately 5%−16% of the evolved strains were identified to be diploid or close to diploid based on our flow cytometry analysis; genomic sequencing and copy number variation (CNV) analyses demonstrated that they were in fact segmental aneuploidies (carrying segmental duplications). The ploidy variations were found in the evolved strains derived from all five parental strains, suggesting that treatment with TBZ can cause ploidy changes in *C. tropicalis* strains of different backgrounds. Since *C. tropicalis* has long been thought to be an obligate diploid organism, we were surprised to find a few potential haploid isolates among our evolved TBZ-resistant strains (Fig 1D). PCR tests demonstrated that both the parental strains E56 and C23 were heterozygous (*MTL***a**/α) at the mating type-like (*MTL*) locus, whereas the derived haploid strains E56-hap only carried the *MTL***a** allele and C23-hap only carried the *MTL*α allele (Fig 1E). The cell sizes of strains E56-hap (2.8–3.5 µm ×  3.4–4.4 µm in diameter) and C23-hap (2.9–4.3 µm ×  3.2–4.7 µm) were much smaller than those of their diploid parental strains (3.8–5.2 µm ×  3.9–6.8 µm for strain E56 and 4.0–5.2 µm ×  4.7–6.4 µm for C23, respectively) (Fig 1F). We next performed whole genome sequencing analysis. CNV and heterozygosity analyses verified the ploidy state of the evolved strains (Figs 2 and S2). As expected, compared to their diploid progenitors, haploid *C. tropicalis* strains exhibited loss of heterozygosity (LOH) at nearly all genomic loci (Fig 2). We noted that extremely few heterozygous sites were observed in the haploid genomes, perhaps due to spontaneous mutations or for technical reasons.

**Fig 3:**
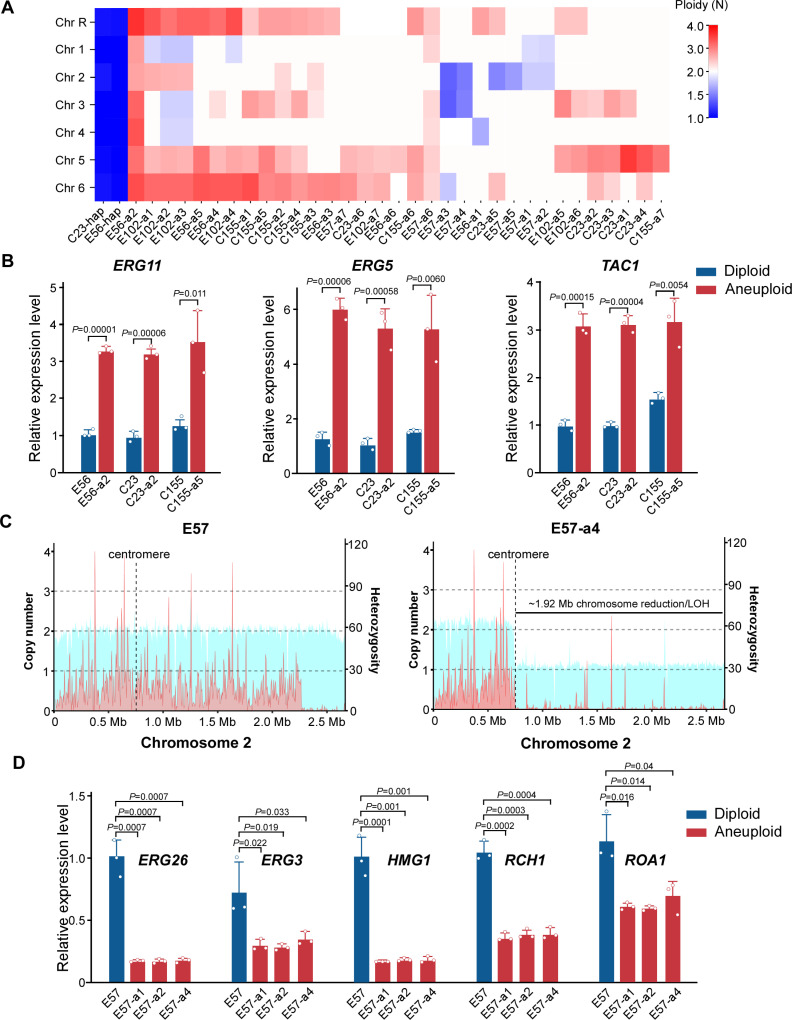
Segmental chromosomal duplication or reduction results in differential expression of resistance-related genes and enhanced azole resistance. (**A**) Chromosomal ploidy levels of the evolved strains. Heatmap indicates the estimated chromosome copy number based on the average coverage level. The names of evolved strains (bottom) and seven chromosomes (left) are shown. (**B**) Relative expression levels of three representative genes with elevated copy number in aneuploid evolved strains. The relative expression levels of *ERG11*, *ERG5*, and *TAC1* were examined using qRT-PCR assays. *ACT1* was used for normalization. (**C**) Copy number distribution (blue area) and heterozygosity level (red area) on chromosome 2 for strain E57 and E57-a4. (**D**) Relative expression levels of five representative genes with reduced copy number in the aneuploid evolved strains: E57-a1, E57-a2, and E57-a4. The diploid progenitor strain E57 served as the control. The data underlying this figure can be found in S3 Data.

Copy number variation analysis revealed that chromosomes 5, 6, and R generally had an increase in copy number, while chromosome 2 had a decrease in copy number (Fig 3A). Notably, segmental duplications of chromosomes 5 and 6 resulted in additional copies of *TAC1*, *ERG5,* and *ERG11*. *TAC1* encodes a transcriptional activator of ABC-transporters, *ERG5* encodes an ergosterol desaturase, and *ERG11* encodes lanosterol demethylase. Additional copies of *ERG11*, *ERG5*, and *TAC1* were found in 20 of our evolved strains. Consistently, Quantitative real-time PCR (qRT-PCR) assays indicated that the relative expression levels of *ERG11*, *ERG5*, and *TAC1* in the azole-resistant strains were significantly increased (Fig 3B). Copy number and heterozygosis analyses indicated that segmental duplication or reduction of chromosomes in the evolved strains tended to occur near or at the centromeres (Figs 3C and S2), which could lead to the formation of isochromosomes. Similar to what has been observed in *C. albicans* [22], the evolved *C. tropicalis* strain with an isochromosome at chromosome 5 exhibited elevated expression levels of *ERG11* and *TAC1* (Fig 3B).

In the evolved strains with a copy number reduction on chromosome 2, we found that several genes located in this region are involved in ergosterol biosynthesis including *HMG1* (Fig 3C). *HMG1* encodes a feedback-sensitive enzyme in the early step of ergosterol synthesis. In *Saccharomyces cerevisiae*, overexpression of *HMG1* leads to a decreased synthesis of ergosterol and hyper-susceptibility to fluconazole, while the dysfunctional or downregulated expression of *HMG1* stimulates cellular ergosterol synthesis and results in an elevated resistance to fluconazole [23]. Moreover, *C. tropicalis ERG3*, *ERG26*, *RCH1*, and *ROA1* genes are also located on the reduced region of chromosome 2. It has been reported that inactivation of these genes in *C. albicans* led to azole resistance [24–27]. As shown in Fig 3D, compared to the progenitor strain, the relative expression levels of these genes were decreased in the evolved *C. tropicalis* strains with a segmental reduction of chromosome 2. Given the critical roles of these genes in azole resistance, changes in copy number could be directly associated with the development of antifungal resistance. Taken together, our findings indicate that exposure to TBZ can induce genomic rearrangements in *C. tropicalis* that result in notable changes in ploidy and cross resistance.

### Haploid *C. tropicalis* strains exhibit reduced growth rates and are attenuated for virulence in a mouse model of systemic infection

**Fig 4:**
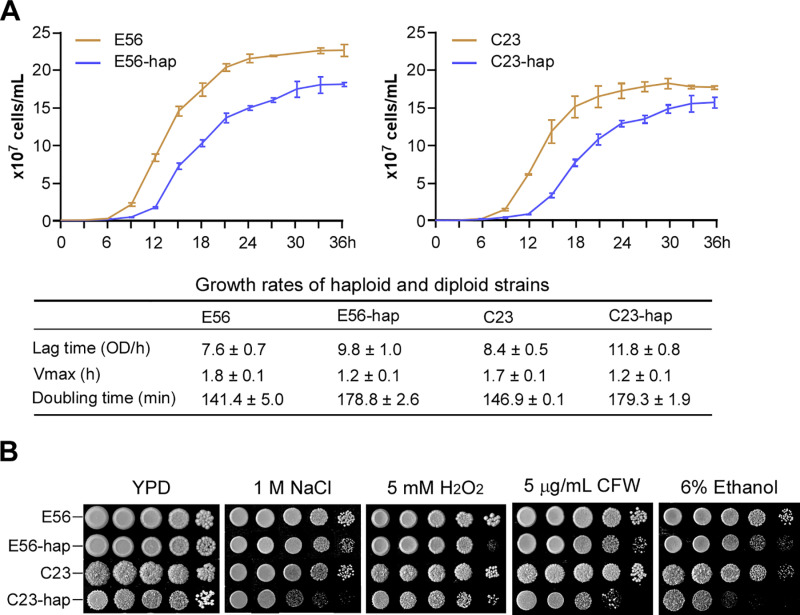
Growth of *Candida tropicalis* haploid and diploid cells under standard and stress conditions. (**A**) Growth curves of haploid and diploid cells. Fungal cells of a single colony were initially grown in liquid YPD medium overnight at 30 °C. Then, 1 × 10^6^ cells were washed twice, re-inoculated into fresh liquid YPD medium, and incubated at 30 °C with shaking (220 rpm). The growth state was monitored by measuring the OD_600_ at the indicated time points. Three biological repeats were performed. The growth curve data were fitted using the DMFit program (https://browser.combase.cc/DMFit.aspx) using default parameters. Doubling times were calculated using the OMNI calculator (https://www.omnicalculator.com/biology/cell-doubling-time). (**B**) Growth of haploid and diploid cells on YPD medium and media containing the indicated chemicals. CFW, calcofluor white. Fungal cells were adjusted to 2 × 10^8^ cells/mL. Tenfold serial dilutions (5 μL) were spotted and grown on the medium at 30 °C for 2 days. The data underlying this figure can be found in S3 Data.

When grown on YPD plates, the evolved haploid *C. tropicalis* strains formed relatively small colonies compared to their diploid counterparts (Fig 1). We therefore examined the growth rates of our evolved haploid and diploid *C. tropicalis* strains in liquid medium. As shown in Fig 4A, both haploid strains (E56-hap and C23-hap) exhibited reduced growth rates in liquid medium and increased doubling times. We next performed serial dilution assays in the presence of several chemical stresses (Fig 4B). On all azole-containing media (TBZ, fluconazole, or voriconazole), the two haploid strains had a notable growth advantage over their diploid progenitors (S1 Fig). However, compared to diploid strains, haploid strains (especially C23-hap) demonstrated growth defects on media containing NaCl, H_2_O_2_, calcofluor white (CFW), or ethanol (Fig 4B), suggesting that diploid *C. tropicalis* cells are more resistant to these environmental stressors.

Since growth rate could be associated with survival in the host, we next compared the virulence of our haploid and diploid *C. tropicalis* strains in a mouse systemic infection model. As expected, compared to haploid cells, diploid cells exhibited increased fungal burden in the mouse (S3A and S3B Fig). Compared to haploid cells, the fungal burdens of diploid cells were significantly higher in the brain, lung, and kidney, but lower in the spleen. Haploid and diploid cells of both background strains (E56 and C23) exhibited overall similar patterns of tissue colonization. The spleen tissue tropism of *C. tropicalis* haploid cells could be associated with their altered pathogenic features such as secretion of virulence factors. Moreover, *C. tropicalis* haploid cells showed a generally higher fungal burden in the tissues compared to diploid cells if the mice were treated with fluconazole prior to fungal cell inoculation (S3C Fig).

### Filamentation, white-opaque switching, and mating in *C. tropicalis* haploid strains

**Fig 5:**
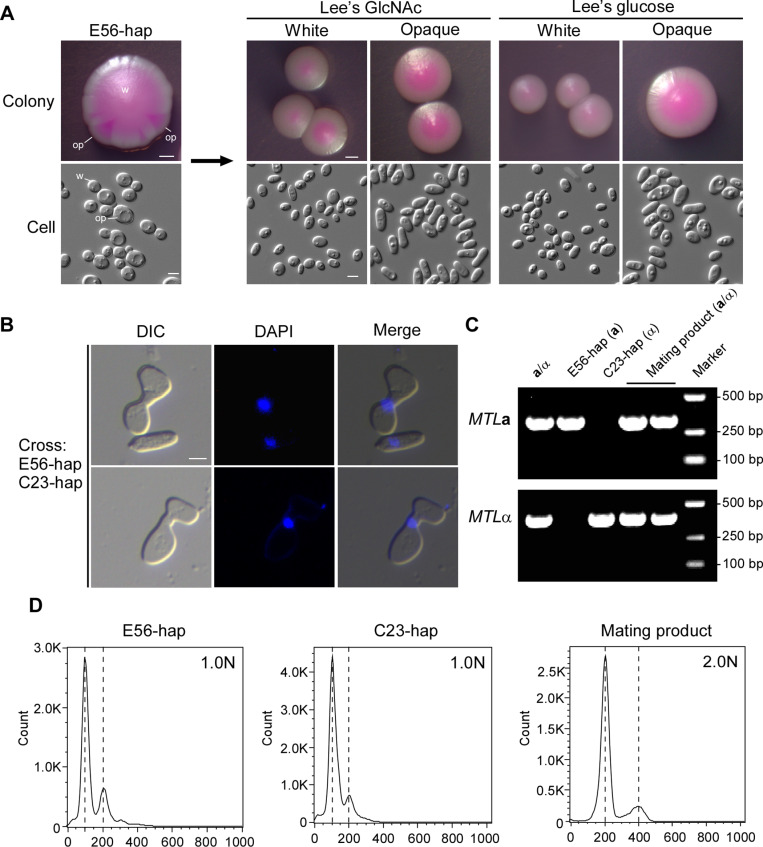
White-opaque transitions and mating between the haploid “a” and “ α” ***Candida tropicalis* strains**. (**A**) Morphologies of colonies and cells of the haploid *C. tropicalis* strains. Cells were initially cultured on SCD medium at 30 °C for 2 days and subsequently replated onto Lee’s GlcNAc medium (pH 6.8, containing 5 μg/mL phloxine B). The plates were incubated at room temperature for 14 days to allow for formation of sectored colonies. The sectors were then replated onto Lee’s GlcNAc and Lee’s Glucose media for 5 days of growth at room temperature. Scale bars: 1 mm for colonies; 5 μm for cells. wh, white; op, opaque. (**B**) Morphology of mating conjugations (stained with DAPI). Approximately 5 × 10^6^ cells of each parental haploid strain (E56-hap and C23-hap) were mixed and spotted on Lee’s GlcNAc medium and grown at 25 °C for 2 days. Cells were collected, washed, and stained using 1 μg/mL 4′,6-diamidino-2-phenylindole (DAPI). DIC, differential interference contrast. (**C**) PCR verification of the *MTL* locus of the parental and mating progeny cells. Strain JX1016 served as the control. (**D**) FACS analysis for the genomic DNA content of the representative mating products and parental strains. The flow cytometry files are available from the Figshare database (https://doi.org/10.6084/m9.figshare.28350296).

The ability to undergo morphological transitions is a critical characteristic of pathogenic *Candida* species [28,29]. We found that haploid *C. tropicalis* strains formed filaments under certain culture conditions, although this ability was weaker than their diploid progenitors (S4 Fig). We also observed that on Lee’s glucose and Lee’s GlcNAc media, haploid *C. tropicalis* strains could undergo white-opaque switching and form elongated opaque cells (Fig 5A). Interestingly, haploid *C. tropicalis* opaque cells were stable not only at 25 °C but also at 37 °C (S5 Fig). These results suggest that like their diploid counterparts, *C. tropicalis* haploid strains are also capable of undergoing white-opaque switching.

Since the white-opaque morphological switch is associated with sexual mating in *C. tropicalis* and *C. albicans* [30–32], we next examined whether *C. tropicalis* haploid cells are mating-competent. When mixed with opaque cells of haploid strains E56-hap (*MTL***a**) and C23-hap (*MTL*α) and cultured on Lee’s GlcNAc medium at 25 °C for 2 days, we observed cell fusion between the cells of opposite mating types. DAPI staining indicated that nuclear fusion occurred (Fig 5B). Heterozygosity at the mating type locus of the mating products was verified by PCR (Fig 5C). FACS analysis demonstrated that the mating progeny had a diploid genome (Fig 5D).

To perform quantitative mating assays, we deleted *ARG4* and *HIS1* genes in haploid strains E56-hap and C23-hap, respectively. Opaque cells of the auxotrophic strains were used for mating assays. The mating efficiency between the *arg4* strain (*MTL***a**) and *his1* strain (*MTL*α) was (7.4 ±  0.8) × 10^−3^ on Lee’s GlcNAc medium, which was slightly lower than that of the diploid cross control perhaps due to the reduced growth rate of haploid cells (S6 Fig). In addition, we also found that *C. tropicalis* haploid opaque cells were able to mate efficiently with progenitor diploid cells to generate triploid mating progenies (S7 Fig). The mating efficiency between the haploid and diploid strains was (3.8 ±  1.1) × 10^−3^ on Lee’s GlcNAc medium. Heterozygosity at the *MTL* locus of the mating products was verified by PCR (S7C Fig). FACS analysis demonstrated that the mating progenies had triploid genomes (S7D Fig). These results suggest that *C. tropicalis* haploid cells can mate with cells that have a variety of ploidy states and mating type statuses.

### *C. tropicalis* haploid and diploid cells differ in global gene expression profiles

**Fig 6:**
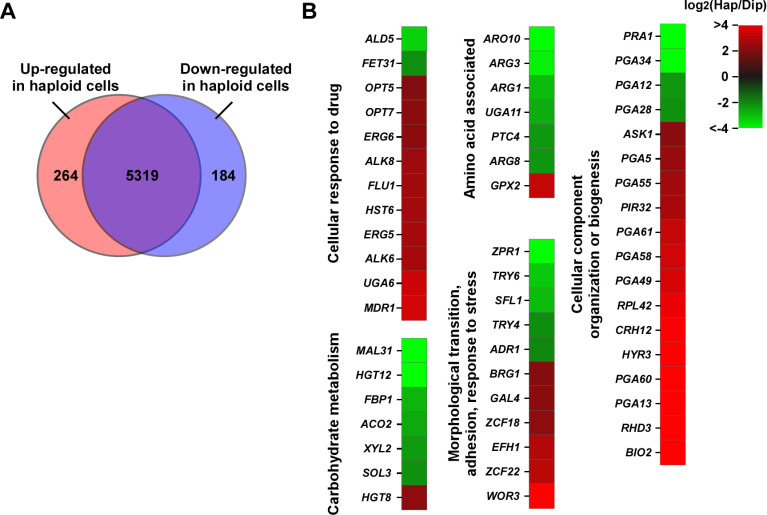
Global gene expression profiles of *Candida tropicalis* haploid (strain E56-hap) and diploid (strain E56) cells. (**A**) Venn diagram of differentially expressed genes (DEGs) defined by a 4-fold change cutoff. (**B**) Heatmap depicting representative DEGs. Hap/Dip, haploid/diploid. The data underlying this figure can be found in S3 Data.

To determine whether these changes in ploidy have an impact on gene expression, as has been well documented in other fungi [20,21,33], we next performed RNA-Seq analysis using haploid and diploid *C. tropicalis* cells (Fig 6 and S2 Data). A total of 448 differentially expressed genes (DEGs) were identified between haploid and diploid cells (using a 4-fold change cutoff). Of them, 264 genes were upregulated in haploid cells and 184 genes were upregulated in diploid cells. These DEGs were associated with several biological processes including nutrient metabolism, stress response, transcriptional regulation, and cell wall integrity. For example, a subset of genes involved in antifungal resistance (e.g., *ERG5* and *ERG6* of the ergosterol biosynthesis pathway; the drug efflux pump-related genes *MDR1*, *FLU1*, and *HST6*; and oligopeptide transmembrane transporter-encoding genes *OPT5* and *OPT7*) were highly expressed in haploid cells (Fig 6B). This expression profile is consistent with the increased antifungal resistance of the evolved haploid strains. Many genes associated with cell wall integrity, adhesion, or morphological transitions were differentially expressed between haploid and diploid cells (e.g., *PGA12*, *PGA25*, *ALS7, BRG1*, *WOR3*, *TRY4*, and *SFL1*), which could contribute to their distinct filamentation abilities and morphological appearances. Moreover, haploid and diploid *C. tropicalis* cells differed in the expression of many metabolic genes. Diploid cells exhibited greater activity in general metabolism than haploid cells, which is consistent with the higher growth rate of diploid cells under standard culture conditions (Figs 4A and 6B). A subset of genes involved in carbohydrate metabolism (e.g., *MAL31*, *HGT12*, *FBP1*, *ACO2*, and *SOL3*) and amino acid metabolism were found to be downregulated in haploid cells. The downregulated genes associated with amino acid biosynthesis included *ARG3*, *ARG1*, and *ARG8* (all involved in arginine biosynthesis) and *UGA11* involved in glutamic acid biosynthesis (Fig 6B and S2 Data). Taken together, the distinct transcriptomic profiles of *C. tropicalis* haploid and diploid cells could contribute to their differences in cell growth, virulence, and fitness under stressful conditions.

### Auto-diploidization of haploid *C. tropicalis* cells

**Fig 7:**
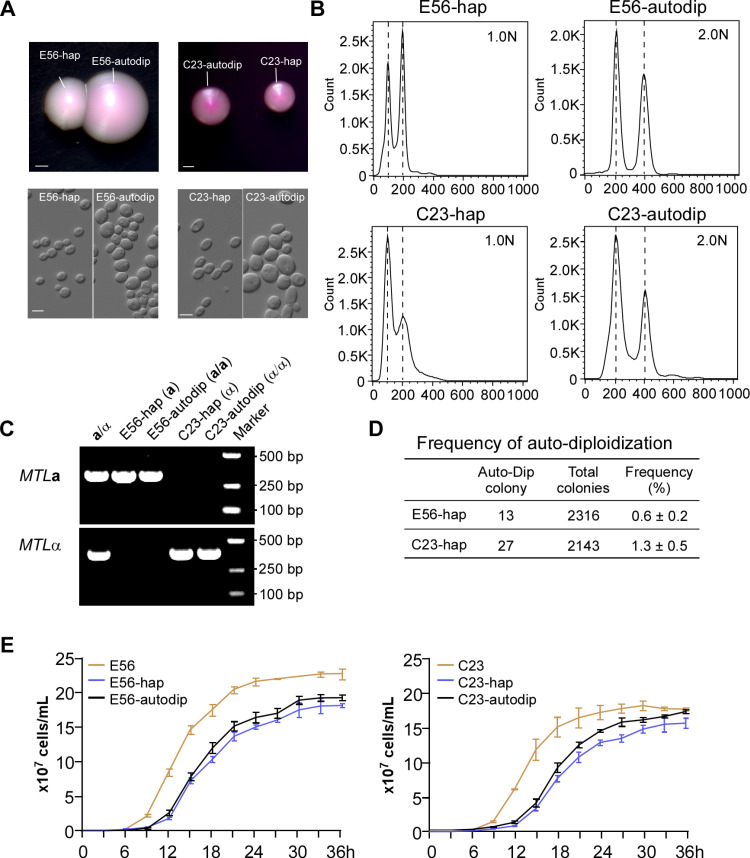
Auto-diploidization of haploid *Candida tropicalis* cells. (**A**) Colony and cellular morphologies of the haploid and auto-diploid strains. Fungal cells were plated onto YPD medium and incubated at 30 °C for 4 days. Scale bars: 1 mm for colonies; 5 μm for cells. (**B**) FACS analysis of the genomic DNA content. (**C**) PCR verification of the *MTL* locus. The *MTL***a**/α diploid (JX1016) strain served as the control. (**D**) Frequency of auto-diploidization. Haploid strains were plated onto YPD medium and incubated at 30 °C for 4 days. Then, all colonies were collected for DNA content analyses using flow cytometry assays. Frequency of auto-diploidization =  total number of diploid colonies/total number of colonies × 100%. Three biological replicates were performed. (**E**) Growth curves. Approximately 1 × 10^6^ cells from a single colony were inoculated into liquid YPD medium and incubated at 30 °C. The growth state was monitored by measuring the OD_600_ at the indicated time points. Three biological replicates of each strain were performed. The data underlying this figure can be found in S3 Data. The flow cytometry files are available from the Figshare database (https://doi.org/10.6084/m9.figshare.28350296).

As seen in haploid *C. albicans* cells [34], haploid *C. tropicalis* cells were unstable and could undergo auto-diploidization and switch to the diploid state at a frequency of 0.6%−1.3% (Fig 7). The colony sizes and morphology of these *C. tropicalis* auto-diploid cells were different from haploid cells. The sizes of the auto-diploid cells were comparable to those of natural diploid cells and were much larger than those of haploid cells (Fig 7A). PCR assays verified that the mating type configuration of the auto-diploids remained unchanged, while their genomic DNA content was doubled as indicated by FACS (Fig 7B and 7C). In liquid medium, the growth rates of the auto-diploids were slightly higher than those of the original haploids but were still much slower than those of the diploid progenitors (Fig 7E). Similar to their haploid progenitors, the auto-diploid strains also exhibited azole resistance. Genomic sequencing analysis indicated that there was a missense mutation G1390A in *ERG11* in the haploid and auto-diploid strains, which leads to a Gly464Ser amino acid substitution. A previous study showed that this Gly464Ser amino acid substitution is associated with fluconazole resistance in *C. tropicalis* [35].

We next examined the stability of haploid strains during systemic infections. As shown in S8 Fig, haploid cells of strain E56-hap were much more stable than those of strain C23-hap after animal passage. Over 90% of the E56-hap cells were maintained in the original haploid state in the five tissues, whereas only 56%−72% of C23-hap cells were maintained in the haploid state after passage through the animal. In addition, when the mice were treated with fluconazole, the auto-diploid strain (E56-autodip) exhibited an enhanced fungal burden compared to the parental diploid strain (E56, S3C Fig).

### Identification of a natural haploid *C. tropicalis* strain

Since azole drugs and environmental fungicides (e.g., TBZ) are widely used in clinical and agricultural settings, respectively, we suspected that haploid *C. tropicalis* strains induced by these chemicals could exist in nature. To test this hypothesis, we revisited the previously published publicly available genomic sequences of 868 *C. tropicalis* strains [10,36,37]. Genome-wide copy number variation and heterozygosity analyses demonstrated that two strains from Spain (ct20 and ct21, NCBI accession numbers SRR12823768 and SRR12823767, respectively) were euploid with extremely low heterozygosity. Similar to strains E56-hap and C23-hap (Fig 2), the genomes of these two strains from Spain underwent extensive (>99%) LOH [10,36], suggesting that strains ct20 and ct21 could be natural *C. tropicalis* haploid or auto-diploid isolates. We obtained strain ct20 from the authors [10] and performed whole genomic sequencing analysis. Consistent with the previous study, the genome exhibited extremely low heterozygosity. We then plated the strain onto YPD medium containing phloxine B and observed the growth of two distinct colonies of different (large and small) sizes (S9 Fig). Flow cytometry analysis indicated that the large and small colonies contained diploid and haploid cells, respectively (S9A Fig). This finding suggests that strain ct20 could be a haploid strain and that a portion of the cells underwent auto-diploidization, perhaps during shipping. A similar phenomenon of auto-diploidization of *C. albicans* haploid cells was previously observed to occur during shipping [34].

## Discussion

Azoles are widely used not only for antifungal therapy in clinical settings but also for crop protection and timber preservation in agricultural settings [38]. Given the wide distribution of human fungal pathogens such as *C. tropicalis, A. fumigatus* and *C. neoformans* in ecological niches, the environmental application and accumulation of azoles could promote the evolution of antifungal resistance in these species in nature. In this study, we report that treatment with the agricultural fungicide TBZ induces ploidy plasticity and the formation of haploid cells in the presumed obligate diploid organism *C. tropicalis*. Aneuploid and haploid descendants of *C. tropicalis* cells treated with TBZ exhibit cross-resistance to fluconazole and voriconazole. Moreover, haploid *C. tropicalis* cells can undergo morphological transitions relevant to mating and are mating competent.

Compared to its closely related species of the *Debaryomycetaceae* clade, such as *C. albicans* and *Candida parapsilosis*, *C. tropicalis* is known to have higher rates of resistance to azole drugs, especially in the Asia-Pacific region [2,3,6]. We recently found that clinical and environmental *C. tropicalis* isolates from China are genetically associated and exhibit high resistance to fluconazole [37], and Chen and colleagues recently reported on the isolation of azole-resistant *C. tropicalis* strains from fruits [39]. Azoles are widely used in agriculture, horticulture, and wood preservation. Agricultural azoles are structurally similar to clinical azoles used in medicine. The occurrence of cross-resistance is not surprising given that both agricultural and clinical azoles target the same ergosterol biosynthesis pathway. Although the maximum residue limit values of TBZ for agricultural use in China are lower than the concentrations of TBZ tested in our study, the final concentration in specific natural niches in the environment could be quite high due to accumulation in certain areas [40]. For example, the concentration of TBZ on plant surfaces, such as leaves, could be significantly increased due to surface evaporation [41]. Moreover, although the residue of TBZ in the soil is likely relatively low, the long-term exposure of *C. tropicalis* cells to the azole could lead to genomic instability. We examined a number of fluconazole-resistant clinical strains and found that all of them showed a cross-resistance to TBZ (MIC ≥ 4 μg/mL). These studies suggest that environmental niches could be a reservoir for drug-resistant fungal pathogens, although it is unclear whether the agricultural use of azoles contributes to the development of antifungal resistance.

Species in the *Debaryomycetaceae* clade such as *C. albicans, C. parapsilosis*, and *C. tropicalis* have long been regarded as “obligate diploid” organisms that carry recessive lethal mutations [34]. Previous studies demonstrated that *C. albicans* can form viable and stable haploid cells during mouse systemic infections or in response to fluconazole in vitro [34,42]. Given the lack of a complete sexual cycle in *C. albicans*, reduction of ploidy could occur through a mechanism of concerted chromosome loss [34]. Fluconazole exposure also induces the formation of drug resistant aneuploids in *C. albicans*, potentially as a result of interrupting cell division [43]. A similar mechanism could be involved in the regulation of ploidy plasticity in *C. tropicalis* upon exposure to environmental azoles. Given that population structures of pathogenic *Candida* species are predominantly clonal in nature and that multiple prerequisites (i.e., *MTL* homozygosis and white-opaque switching) are needed for mating, ploidy transitions induced by stress may represent an alternative strategy of generating genetic diversity other than sexual reproduction. However, it remains unclear how haploid cells are formed in these pathogenic species and what advantages these evolved haploids have over their diploid counterparts in nature.

We observed that haploid *C. tropicalis* cells could spontaneously switch to the diploid form (auto-diploidization). Homozygous diploid cells generated from haploid cells exhibited a slightly increased cell growth rate compared to haploid cells, although this rate was still much slower than that of their diploid progenitors (Fig 7). This finding suggests that auto-diploidization is unable to fully recover the loss of fitness that haploid *C. tropicalis* cells experience, perhaps due to the loss of some genes or heterozygosity.

Like *C. albicans* [34]*, C. tropicalis* haploid cells grow slower and are less virulent than diploid cells (Figs 4 and S3). We previously demonstrated that *Candida glabrata* and *Candida auris* are also able to undergo changes in ploidy. Although the haploid state had been thought to be the baseline ploidy, haploid cells of *C. glabrata* and *C. auris* exhibit a reduced virulence compared to their diploid counterparts [20,21], which is consistent with the virulence patterns we observed in *C. tropicalis*. Moreover, the reduced fitness and growth rates observed in *C. tropicalis* haploid strains could also be a consequence of activation of recessive alleles. Global transcriptional expression profiling analysis demonstrates that DEGs of haploid and diploid *C. tropicalis* cells are involved in several biological processes including nutrient metabolism, response to stresses, and cell wall integrity, suggesting that ploidy changes have a considerable impact on cellular physiology.

Morphological transition analyses demonstrate that haploid *C. tropicalis* cells can form filaments and undergo white-opaque switching (S4 and S5 Figs). Opaque cells of the haploid strains can mate with haploid or progenitor diploid cells of the opposite mating type, although the mating efficiencies were slightly reduced compared to those of the “diploid × diploid” cross controls perhaps due to their slower cell growth rates. These results suggest that a single set of chromosomes is sufficient for morphological transitions and mating in *C. tropicalis*.

## Conclusions

Fungal pathogens display genomic plasticity that is important for the development of antifungal resistance and for survival under changing environmental conditions. Excessive use of azole fungicides in agriculture, antifouling coatings, and wood preservatives could have a considerable impact on the environment. Their contamination of water and soil could lead to the evolution of drug resistance in fungal pathogens of both plants, animals, and humans. Here we report that exposure to the widely used agricultural fungicide TBZ results in the formation of drug resistant aneuploid and haploid *C. tropicalis* cells, which exhibit cross-resistance to azoles used in clinical settings. Importantly, the TBZ-induced *C. tropicalis* haploid cells were able to undergo mating and phenotypic switching. Furthermore, genomic analysis of a clinical *C. tropicalis* strain indicates its extremely low heterozygosity and flow cytometry assays verify its haploid character, suggesting that haploidization could occur in nature. Given the wide distribution of *C. tropicalis* in ecological niches, environmental azole-induced genomic plasticity could represent an evolutionary mechanism that promotes genetic diversity. Our study suggests that there could be a link between the increased number of infections caused by azole-resistant *C. tropicalis* isolates in clinical settings and the widespread use of environmental azoles. Moreover, the discovery of stable *C. tropicalis* haploid cells could provide the field with the framework for a tractable tool for future genetic analyses, especially for the study of recessive alleles in this important fungal pathogen.

## Materials and methods

### Ethics statement

All animals received humane care in compliance with general guidelines approved by the Animal Care and Use Committee of the School of Life Science at Fudan University (2021JS004). The present study was approved by the Committee.

### Strains and culture conditions

*C. tropicalis* strains used in this study are listed in S1 Table. *C. tropicalis* cells were routinely cultured in YPD medium (10 g/L yeast extract, 20 g/L peptone, 20 g/L glucose; 20 g/L agar was added for solid medium) and SCD (synthetic complete dextrose) medium. RPMI-1640 (Thermo Fisher Scientific) with MOPS (morpholinepropanesulfonic acid) medium containing 20 g/L glucose was used for in vitro experimental evolution assays. Modified Lee’s glucose and Lee’s GlcNAc media [44] were used for morphological analysis and mating assays. For quantitative mating assays, SCD amino acid dropout media were used for selectable growth. Phloxine B, a red dye, was added to the media to distinguish *C. tropicalis* colonies with an altered ploidy or white/opaque colonies. This dye has been widely used for the studies of ploidy shift in several yeast species [20,21]. All strains were stored at −80 °C in 25% glycerol.

### Antifungal susceptibility test

Antifungal susceptibility tests were performed using a broth microdilution method as described in the Clinical and Laboratory Standards Institute (CLSI) M27 guidelines [45]. Antifungals were purchased from Sigma-Aldrich (St. Louis, Missouri, USA) and dissolved in DMSO (Dimethyl sulfoxide). The concentration ranges were 0.06–64 μg/mL for TBZ and voriconazole, 0.125–128 μg/mL for fluconazole, and 0.03–32 μg/mL for itraconazole, posaconazole, caspofungin, and amphotericin B. *Candida krusei* ATCC 6258 and *Candida parapsilosis* ATCC 22019 served as quality controls. The MIC_50_ was defined as the minimal concentration at which 50% of growth was inhibited (relative to the control). A provisional breakpoint for TBZ was set to 2 μg/mL in this study based on the susceptibility distribution of this species.

### In vitro experimental evolution assay

A schematic overview of the experimental evolution assay is presented in Fig 1A. Five *C. tropicalis* isolates (three environmental isolates E56, E57, and E102 and two clinical isolates C23 and C155) were used as starting strains. *C. tropicalis* cells were initially grown on YPD agar and incubated at 30 °C for 48 h. A single colony of each strain was inoculated into RPMI-1640 with MOPS medium (with 2% glucose) and incubated overnight at 37 °C. Subsequently, 1 × 10^6^ cells were diluted into 5 mL of fresh RPMI-1640 with MOPS medium with no drug (control) or a concentration of 2 × MIC_50_ TBZ. The cultures were incubated at 37 °C for 3 days in a shaking incubator. After 3 days of incubation, 0.5 mL of the culture was transferred into 4.5 mL of fresh medium containing the same concentration of TBZ as the previous culture. Three passages were performed under the same culture conditions. Afterwards, the TBZ concentration was doubled for subsequent passages. This stepwise increase in the TBZ concentration was repeated until a final concentration of 16 μg/mL was reached. When the experimental evolution process was completed, the final cultures were plated onto YPD plates containing 16 μg/mL TBZ and incubated at 37 °C for 4 days. Single colonies were picked from the plates for further analysis.

### Flow cytometry analysis

The genomic DNA content of *C. tropicalis* was determined by FACS analysis as described previously with slight modifications [20]. Cells of single colonies were incubated in liquid YPD medium at 30 °C and grown to exponential phase. Fungal cells were then harvested, washed with double-distilled water, and resuspended in 300 μL TE buffer (10 mM Tris, 1 mM EDTA, pH 8.0) and mixed with 700 μL 100% ethanol in RNase-free tubes. The samples were placed on a vortex mixer (Vortex-Genie 2) for 3 h. Cells were collected, washed, and resuspended with 1 mL TE buffer, and then incubated with RNase A (0.3 mg/mL) overnight at 37 °C. The samples were further treated with proteinase K (0.03 mg/mL) and incubated for 1 h at 50 °C. Fungal cells were then collected, washed twice with TE buffer, suspended in phosphate-buffered saline (PBS), and stained with propidium iodide (25 μg/mL) for genomic DNA content analyses. At least 30,000 cells were detected on a BD FACSCalibur system and the data were analyzed using the software FlowJo 10.4.

### Whole-genome sequencing and genomic analysis

Single colonies of *C. tropicalis* were grown overnight in SCD medium at 30 °C. Cells were collected for genomic DNA extraction using the TIANamp Yeast DNA Kit (Cat. No. DP307-02) according to the manufacturer’s protocol. The construction of libraries, whole-genome sequencing, and raw data processing were performed by BGI (Wuhan, China). Methods for genome analysis were performed as described in our previous publication [46]. Briefly, clean reads were mapped to the genomic assembly of *C. tropicalis* strain MYA-3404 (NCBI accession number: GCA_013177555.1) by BWA mem v0.7.17. SAMTools v1.12, Picard Tools v1.56 (http://picard.source-forge.net), and Genome Analysis Toolkit (GATK) v3.4 were used for variant calling. To present the distribution of heterozygosity, the number of heterozygous sites per 5,000 bp was calculated using bcftools v1.9. The copy number variation across genomic regions was identified based on the read depth of 1,000-bp nonoverlapping windows using the Splint script to avoid the “smiley pattern” bias, and visualized using R script.

### RNA-Seq analysis

The *C. tropicalis* haploid strain E56-hap and the diploid strain E56 were used for global transcriptional profiling experiments. Fungal cells were incubated in liquid SCD medium at 30 °C for 8 h. FACS analysis was performed to ensure their ploidy state before extraction. Total RNA of fungal cells was extracted using the GeneJET RNA Purification Kit (Thermo Scientific) according to the manufacturer’s instructions. RNA sequencing was conducted using the Illumina NovaSeq 6000 (Berry Genomics Co, Beijing). Clean reads were aligned to the genomic assembly of *C. tropicalis* strain MYA-3404 (NCBI accession number: GCA_013177555.1) by HiSat2 v2.0.5. Transcriptional expression of different strains was estimated by StringTie v1.3.3b. DEGs (fold change ≥  4, FDR ≤  0.01) were identified using the DESeq2 R package. The expression levels of the diploid strain E56 served as the reference.

### Quantitative real-time PCR assay (qRT-PCR)

A total of 1.0 μg of RNA was used as the template to synthesize cDNA with the RevertAid H Minus Reverse Transcriptase (Thermo Scientific). qRT-PCR was performed with a Bio-Rad CFX96 real-time PCR system using a SYBR green mix (TOYOBO). The relative expression levels of target genes were normalized to the *C. tropicalis ACT1* gene. Each experiment was performed in three biological replicates. All the primers used in this study are listed in S2 Table.

### Fungal burden assays and ploidy stability in a murine host

In vivo assessment of fungal burdens was carried out using an established murine systemic infection model as described previously with slight modifications [20]. Briefly, fungal cells of diploid and haploid strains were initially grown on YPD medium at 30 °C for 2 days. Cells were collected and washed three times with 1 × PBS. 6–8-week-old female BALB/c mice (*N* =  5 per strain) were inoculated intravenously with 1 × 10^7^ cells in 200 μL 1 × PBS. After 24 h of infection, the CFUs in the brain, liver, spleen, lung, and kidney were evaluated by plating the tissue homogenates onto YPD plates (500–1,000 CFUs/plate). To assess the survival rate of *C. tropicalis* cells in the mice treated with fluconazole, 100 μg fluconazole in 200 μL PBS (with final concentration of 5 mg/kg) was injected intraperitoneally into each mouse 30 min prior to infection. The animals injected with 200 μL PBS served as the controls. 1 × 10^7^ *C. tropicalis* cells were injected into the mouse through the tail vein. Fungal cells were collected and plated onto YPD plates after 24 h of infection for fungal burden analysis. Survival rate (%) =  Number of CFUs (with fluconazole treatment group)/Number of CFUs (without fluconazole treatment group) × 100%.

To evaluate the stability of the haploids, the genomic DNA content of the *C. tropicalis* cells recovered from different animal tissues was determined by flow cytometry analysis.

### Construction of *C. tropicalis* gene knockout mutant strains

A schematic overview of the “one-step” gene deletion in haploid *C. tropicalis* strains is shown in S6A Fig. Primers used are listed in S2 Table. To construct *ARG4* and *HIS1* plasmids pSFS2A-*ARG4*-KO and pSFS2A-*HIS1*-KO, the fragments of the 5′- and 3′-fragments of *ARG4* and *HIS1* were amplified from the genomic DNA of *C. tropicalis* by PCR and inserted into the *Apa*I/*Xho*I and *Sac*II/*Sac*I sites of the plasmid pSFS2A [47]. To generate the *arg4* and *his1* mutant strains, the plasmids were linearized with *Apa*I/*Sac*I and transformed into the haploid strains.

### Quantitative mating assays

*C. tropicalis* mating assays were performed as described in our previous publication [48]. Briefly, strains were initially grown on Lee’s GlcNAc medium at 25 °C for 7 days. Homogeneous opaque cells were collected and resuspended in ddH_2_O at a concentration of 1 × 10^9^ cells/mL. Two parental strains (5 μL for each) were mixed and spotted onto Lee’s GlcNAc medium and incubated at 25 °C for 7 days. Subsequently, mating mixtures were collected and plated onto SCD-His, SCD-Arg, and SCD-Arg-His dropout media for prototrophic selection growth. Mating efficiencies were calculated based on the CFUs from the three types of plates.

## Supporting information

S1 Fig Growth of the wildtype and evolved strains on YPD medium containing azole drugs. *Candida tropicalis* cells were adjusted to 2 ×  10^8^ cells/mL and then 10-fold serial dilutions of cells were spotted onto YPD or YPD medium containing 16 μg/mL tebuconazole, 16 μg/mL fluconazole, or 2 μg/mL voriconazole. The plates were incubated at 37 °C for 2 days. TBZ, tebuconazole; FLC, fluconazole; VOC, voriconazole.
(TIF)

S2 Fig Copy number distribution of *Candida tropicalis* strains after a series of passages in tebuconazole containing medium. Diploid strain C23 served as a reference. The *x*-axis indicates chromosomes R through 6. The *y*-axis represents the copy number of each chromosome. Each point indicates an average copy number of a genomic segment of 1,000 bp across the genome based on the coverage analysis of the genomic data. The blue dashed lines indicate the positions of the centromere.
(TIF)

S3 Fig Fungal burdens of *Candida tropicalis* haploid and diploid strains in a mouse systemic infection model. (**A**) Strains E56 and E56-hap. (**B**) Strains C23 and C23-hap. Quantitative fungal burdens of the brain, liver, lung, spleen, and kidney were examined. After 24 h of infection, the fungal burdens were assessed in the five organs. Five 6–8-week-old BALB/c mice were used for each strain. **p <  0.01, NS, no significance (two-tailed Student *t* test). (**C**) The survival rates of haploid, diploid, and auto-diploid cells of *C. tropicalis* strains in different mouse tissues in a systemic infection model. The mice were treated with 5 mg/kg fluconazole prior to inoculation. Haploid (E56-hap), diploid (E56), and auto-diploid (E56-autodip) cells were grown on YPD medium at 30 °C for 2 days. Fungal cells were collected and suspended in PBS and used for injection. 100 μg fluconazole in 200 μL PBS (with final concentration of 5 mg/kg) was injected intraperitoneally into the mouse, and 200 μL PBS served as the reference. After 30 min of drug treatment, a 200 μL suspension containing 1 ×  10^7^ cells was injected into each mouse via the tail vein. Four mice were used for each group. After 24 h of infection, the brain, liver, spleen, lung, and kidney were analyzed for fungal burdens. Survival rate (%) =  Number of CFUs (with fluconazole treatment group)/Number of CFUs (without fluconazole treatment group) × 100%. The data underlying this figure can be found in S3 Data.
(TIF)

S4 Fig Filamentous growth of *Candida tropicalis* haploid and diploid strains. Fungal cells were plated onto Lee’s glucose medium and incubated at 37 °C for 5 days. Scale bars: 1 mm for colonies, 5 μm for cells.
(TIF)

S5 Fig Haploid white and opaque cells at 37 °C. Fungal cells of haploid strain E56-hap were plated onto Lee’s glucose and Lee’s GlcNAc medium, and incubated at 37 °C for 3 days. Scale bars: 1 mm for colonies, 5 μm for cells.
(TIF)

S6 Fig One-step gene deletion strategy and quantitative mating assays in haploid *C. tropicalis* strains. (**A**) Schematic diagram of gene deletion strategy based on homologous recombination (exemplified by the gene *ARG4*). The *caSAT1*-flipping cassette was used for *ARG4* deletion. *caFLP*, *Candida*-adapted *FLP* gene; P_*MAL2*_, *MAL2* promoter; FRT, *FLP* recombination target; *caSAT1*, *Candida*-adapted *SAT1* marker; T_*ACT1*_, transcription termination sequence of the *ACT1* gene. (**B**) Cellular morphology of the auxotrophic haploid strains (E56-hap, *arg4*; C23-hap, *his1*). Scale bar, 5 μm. Op, opaque. (**C**) Mating growth of haploid strains. Haploid parental strains: E56-hap (*MTL***a**, *arg4*) and C23-hap (*MTL*α, *his1*). Haploid cells and their mixture were grown on the three types of selective SCD media lacking corresponding amino acids. (**D**) Mating efficiencies of haploid and diploid strains on Lee’s GlcNAc at 25 °C for 7 days. The data underlying this figure can be found in S3 Data.
(TIF)

S7 Fig Mating between haploid and diploid strains of *Candida tropicalis.
* (**A**). Mating growth of parental and mixture cells. Haploid parental strain, E56-hap (*MTL***a**, *arg4*); diploid parental strain, CAY2061 (*MTL*α/α, *his1*/*his1*). Parental cells were mixed and grown on Lee’s GlcNAc at 25 °C for 7 days, and their mixtures were grown on the three types of selective SCD media lacking corresponding amino acids. (B) Morphology of mating conjugations. Approximately 5 × 10^6^ cells of each parental strain were mixed and spotted onto Lee’s GlcNAc medium and grown at 25 °C for 2 days. Scale bar, 5 μm. (**C**) PCR verification of the *MTL* locus of the parental and mating product. (**D**) FACS analysis for the genomic DNA content of the representative mating product and parental strains. (**E**) Mating efficiency between the haploid and diploid strains on Lee’s GlcNAc at 25 °C for 7 days. The data underlying this figure can be found in S3 Data. The flow cytometry files are available from the Figshare database (https://doi.org/10.6084/m9.figshare.28350296).
(TIF)

S8 Fig Ploidy conversion of colonies isolated from mice systemically infected with haploid *Candida tropicalis* strains. (**A**) Schematic of the ploidy stability test using the mouse infection model. (**B**) Percentages of the haploid and auto-diploidization colonies isolated from the mice systemically infected with strain E56-hap. (**C**) Percentages of the haploid and auto-diploidization colonies isolated from the mice systemically infected with strain C23-hap. Approximately 1 × 10^7^ haploid cells in 200 μL PBS were injected into mouse tail veins; fungal cells were recovered from the liver, kidney, brain, lung, and spleen 24 h post-infection, and replated onto YPD medium containing 5 μg/mL phloxine B. Ploidy states of all distinct colonies were inferred by FACS analysis. Bar plots represent the ploidy ratios of colonies isolated from the mice. The data underlying this figure can be found in S3 Data.
(TIF)

S9 Fig Discovery of the natural haploid *Candida tropicalis* isolate ct20. (**A**) Morphology of the natural haploid and auto-diploid strains. Fungal cells were plated onto YPD medium containing 5 µg/mL phloxine B and cultured at 30 °C for 3 days. Two distinct colonies were then replated onto a fresh YPD medium with phloxine B and cultured under the same condition. Scale bars for colonies and cells were 1 mm and 5 μm, respectively. (**B**) Scatter plot of the genome-wide copy number distribution. The *x*-axis represents the seven chromosomes and the *y*-axis represents the relative copy numbers. Each point indicates an average copy number of a genomic segment of 1,000 bp across the genome based on the coverage analysis of the genomic data. The flow cytometry files are available from the Figshare database (https://doi.org/10.6084/m9.figshare.28350296).
(TIF)

S1 Data Antifungal profiles of the *Candida tropicalis* isolates used in this study. (XLSX)

S2 Data RNA-Seq analysis for the *Candida tropicalis* haploid and diploid strains. (XLSX)

S3 Data Underlying data for the figures. (XLSX)

S1 Table Strains used in this study. (DOCX)

S2 Table Primers used in this study. (DOCX)

## Abbreviations

CNV: copy number variation
DEGs: differentially expressed genes
LOH: loss of heterozygosity
*MTL*: mating type-like
PBS: phosphate-buffered saline
qRT-PCR: Quantitative real-time PCR
TBZ: tebuconazole
