# Supplementary figures and images for: An agricultural triazole induces genomic instability and haploid cell formation in the human fungal pathogen Candida tropicalis
Source: PLoS Biol. 2025 Apr 1;23(4):e3003062. doi: 10.1371/journal.pbio.3003062 (PMC11960876; doi:10.1371/journal.pbio.3003062)

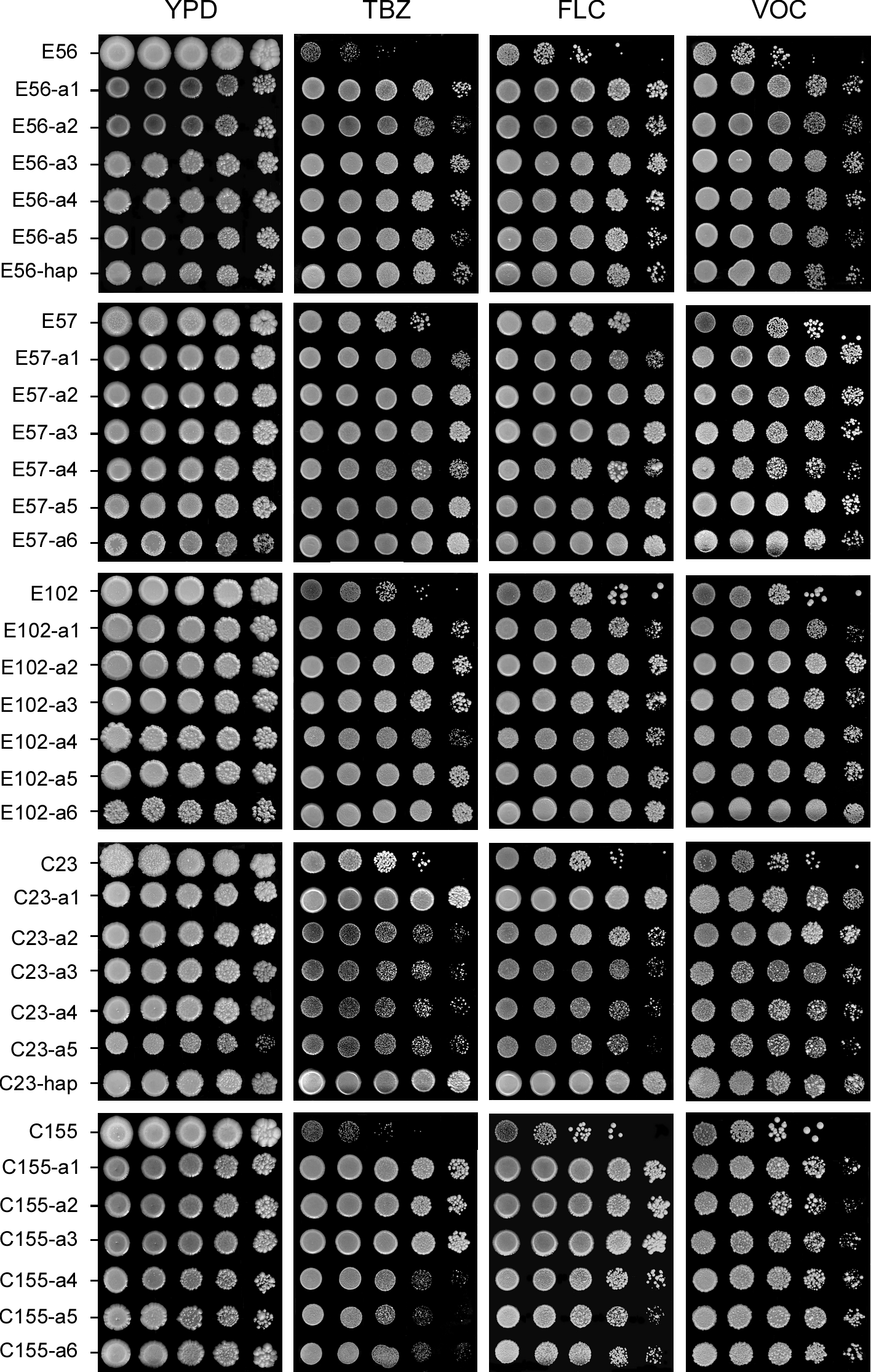

Supplement: S1 Fig — Candida tropicalis cells were adjusted to 2 × 10^8^ cells/mL and then 10-fold serial dilutions of cells were spotted onto YPD or YPD medium containing 16 μg/mL tebuconazole, 16 μg/mL fluconazole, or 2 μg/mL voriconazole. The plates were incubated at 37 °C for 2 days. TBZ, tebuconazole; FLC, fluconazole; VOC, voriconazole. (TIF) [file pbio.3003062.s001.tif]

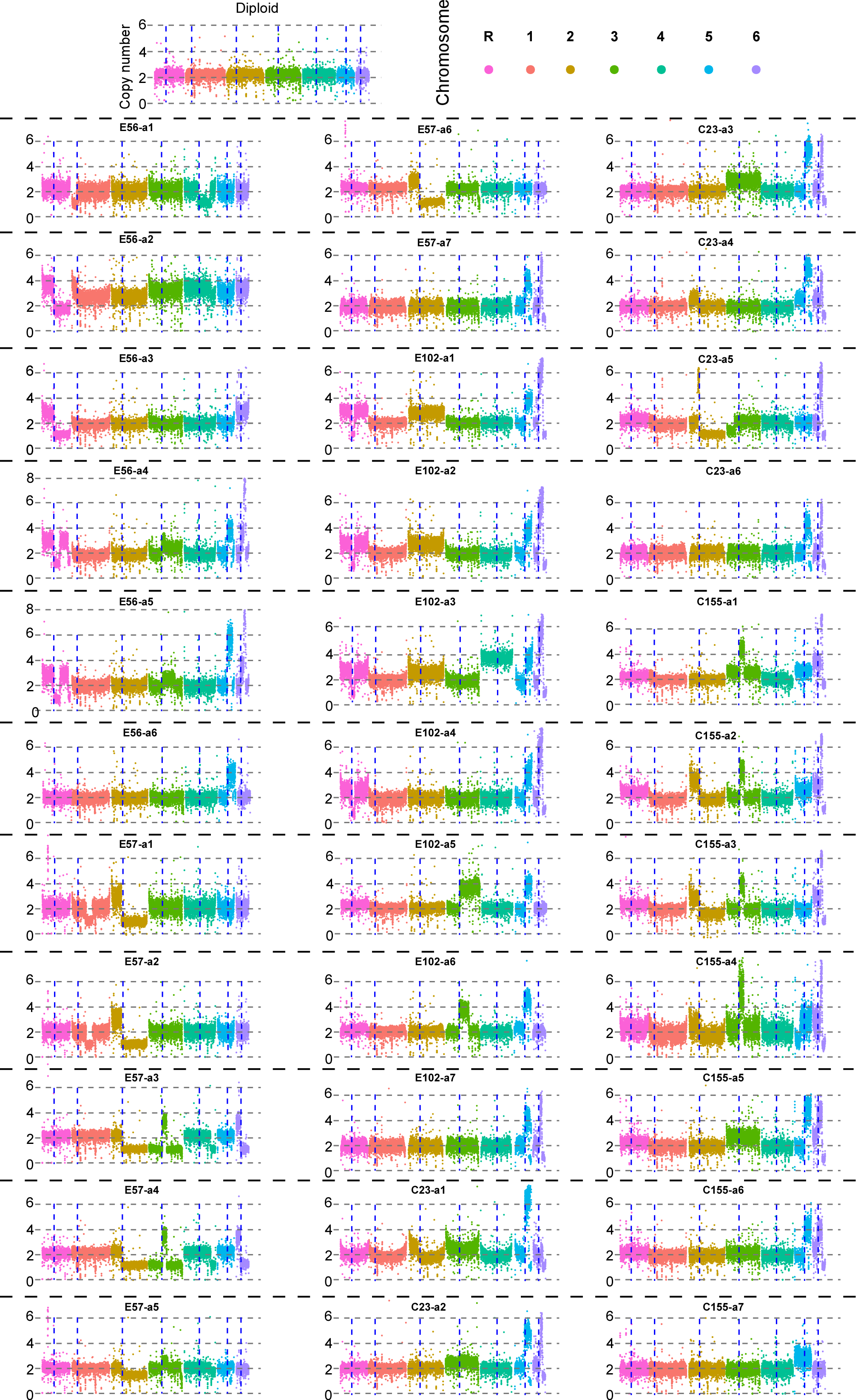

Supplement: S2 Fig — Diploid strain C23 served as a reference. The x-axis indicates chromosomes R through 6. The y-axis represents the copy number of each chromosome. Each point indicates an average copy number of a genomic segment of 1,000 bp across the genome based on the coverage analysis of the genomic data. The blue dashed lines indicate the positions of the centromere. (TIF) [file pbio.3003062.s002.tif]

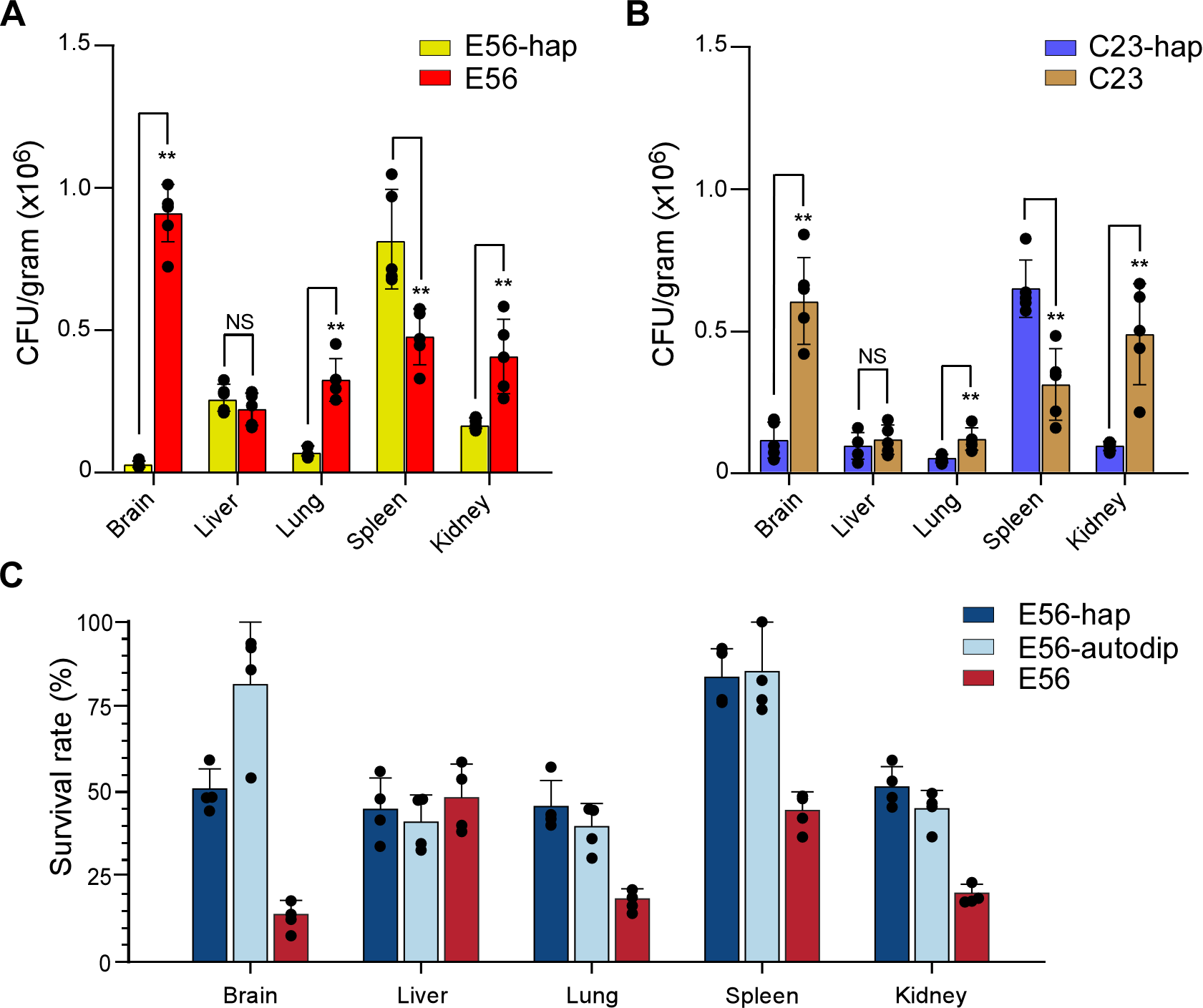

Supplement: S3 Fig — (A) Strains E56 and E56-hap. (B) Strains C23 and C23-hap. Quantitative fungal burdens of the brain, liver, lung, spleen, and kidney were examined. After 24 h of infection, the fungal burdens were assessed in the five organs. Five 6–8-week-old BALB/c mice were used for each strain. **p < 0.01, NS, no significance (two-tailed Student t test). (C) The survival rates of haploid, diploid, and auto-diploid cells of C. tropicalis strains in different mouse tissues in a systemic infection model. The mice were treated with 5 mg/kg fluconazole prior to inoculation. Haploid (E56-hap), diploid (E56), and auto-diploid (E56-autodip) cells were grown on YPD medium at 30 °C for 2 days. Fungal cells were collected and suspended in PBS and used for injection. 100 μg fluconazole in 200 μL PBS (with final concentration of 5 mg/kg) was injected intraperitoneally into the mouse, and 200 μL PBS served as the reference. After 30 min of drug treatment, a 200 μL suspension containing 1 × 10^7^ cells was injected into each mouse via the tail vein. Four mice were used for each group. After 24 h of infection, the brain, liver, spleen, lung, and kidney were analyzed for fungal burdens. Survival rate (%) = Number of CFUs (with fluconazole treatment group)/Number of CFUs (without fluconazole treatment group) × 100%. The data underlying this figure can be found in S3 Data. (TIF) [file pbio.3003062.s003.tif]

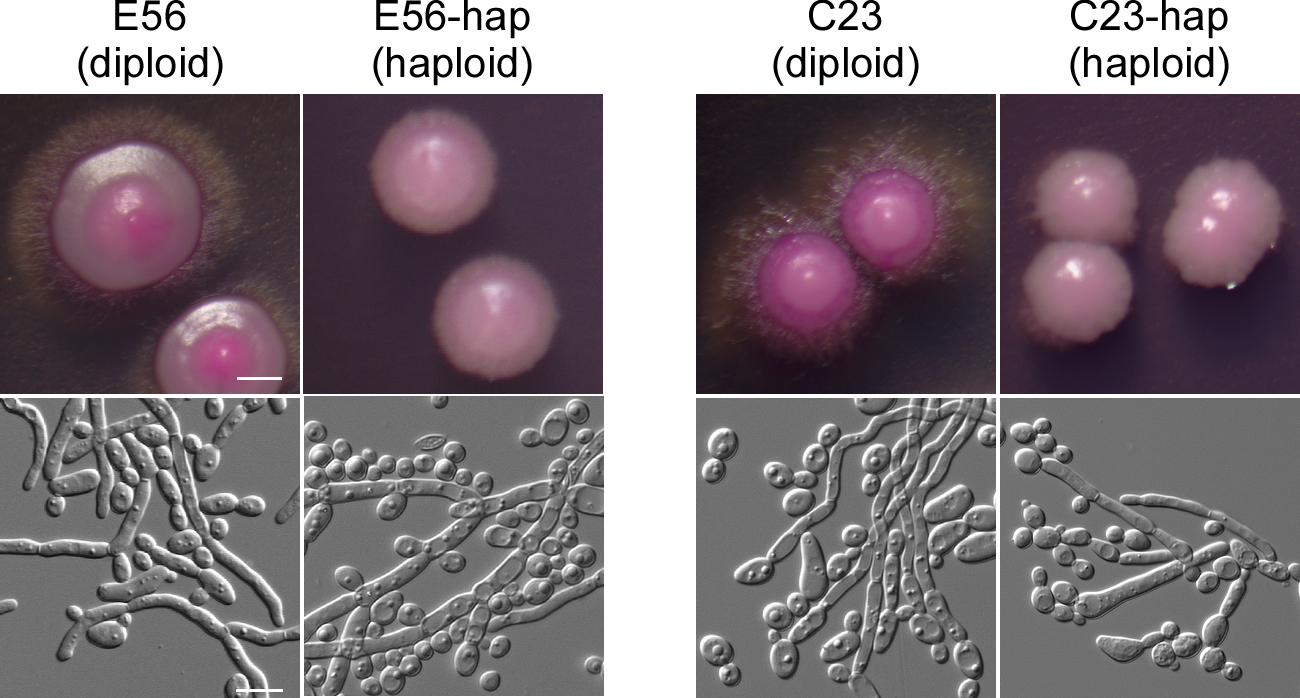

Supplement: S4 Fig — Fungal cells were plated onto Lee’s glucose medium and incubated at 37 °C for 5 days. Scale bars: 1 mm for colonies, 5 μm for cells. (TIF) [file pbio.3003062.s004.tif]

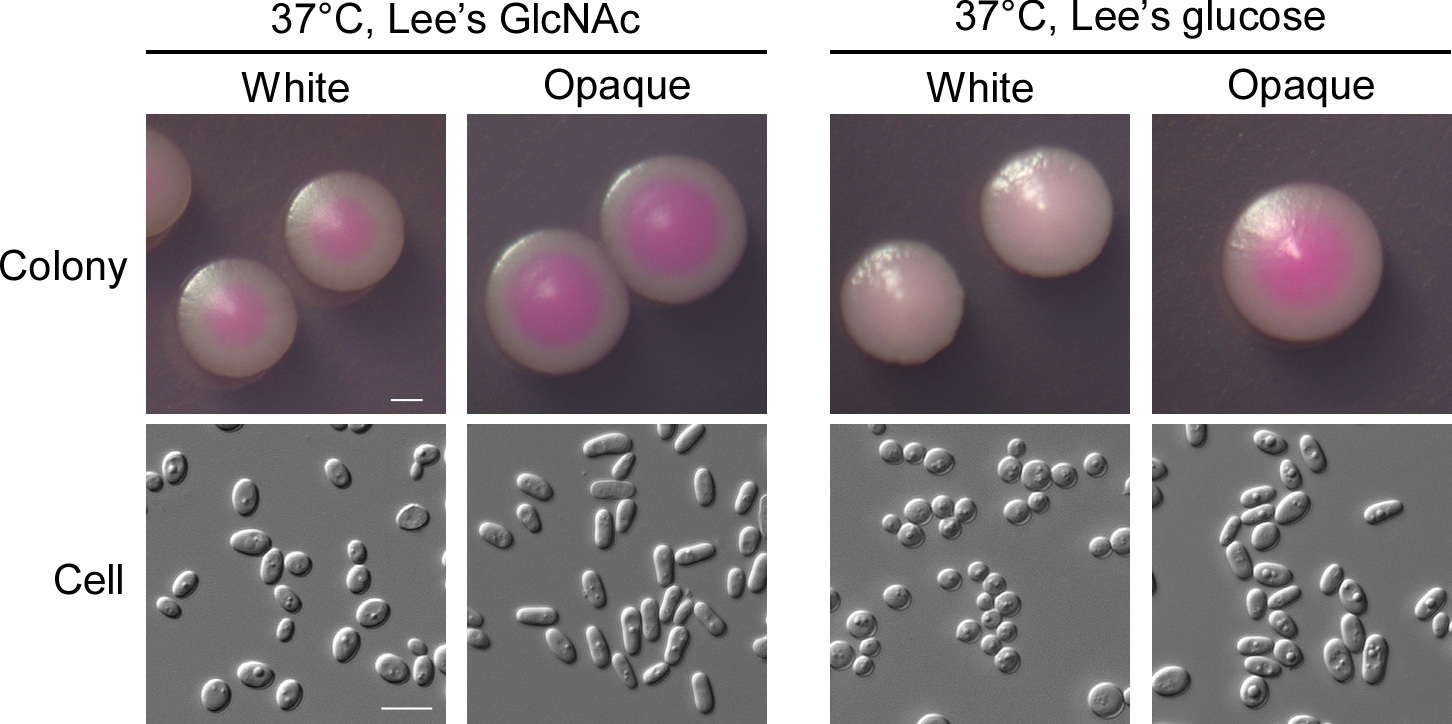

Supplement: S5 Fig — Fungal cells of haploid strain E56-hap were plated onto Lee’s glucose and Lee’s GlcNAc medium, and incubated at 37 °C for 3 days. Scale bars: 1 mm for colonies, 5 μm for cells. (TIF) [file pbio.3003062.s005.tif]

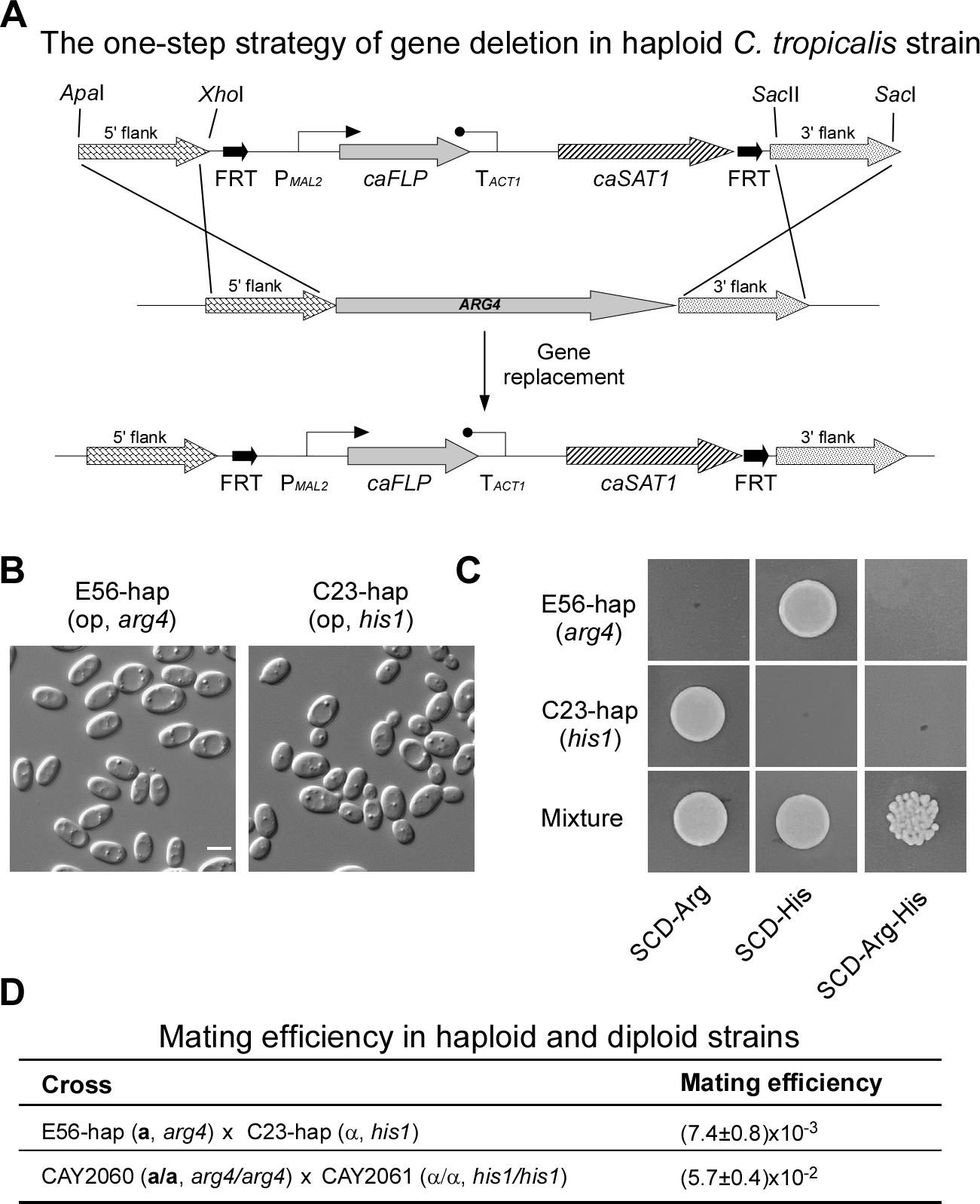

Supplement: S6 Fig — (A) Schematic diagram of gene deletion strategy based on homologous recombination (exemplified by the gene ARG4). The caSAT1-flipping cassette was used for ARG4 deletion. caFLP, Candida-adapted FLP gene; PMAL2, MAL2 promoter; FRT, FLP recombination target; caSAT1, Candida-adapted SAT1 marker; TACT1, transcription termination sequence of the ACT1 gene. (B) Cellular morphology of the auxotrophic haploid strains (E56-hap, arg4; C23-hap, his1). Scale bar, 5 μm. Op, opaque. (C) Mating growth of haploid strains. Haploid parental strains: E56-hap (MTLa, arg4) and C23-hap (MTLα, his1). Haploid cells and their mixture were grown on the three types of selective SCD media lacking corresponding amino acids. (D) Mating efficiencies of haploid and diploid strains on Lee’s GlcNAc at 25 °C for 7 days. The data underlying this figure can be found in S3 Data. (TIF) [file pbio.3003062.s006.tif]

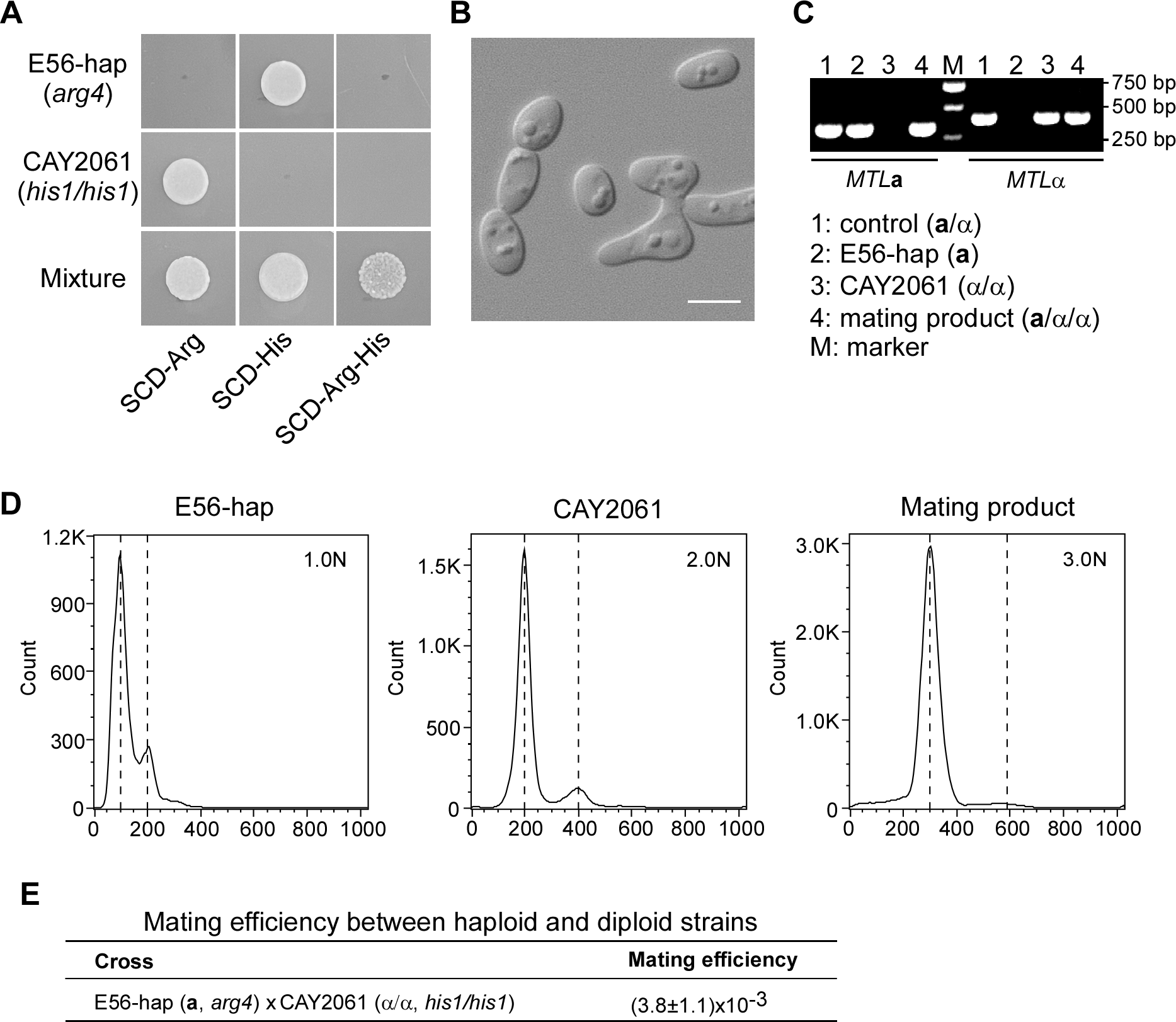

Supplement: S7 Fig — (A). Mating growth of parental and mixture cells. Haploid parental strain, E56-hap (MTLa, arg4); diploid parental strain, CAY2061 (MTLα/α, his1/his1). Parental cells were mixed and grown on Lee’s GlcNAc at 25 °C for 7 days, and their mixtures were grown on the three types of selective SCD media lacking corresponding amino acids. (B) Morphology of mating conjugations. Approximately 5 × 10^6^ cells of each parental strain were mixed and spotted onto Lee’s GlcNAc medium and grown at 25 °C for 2 days. Scale bar, 5 μm. (C) PCR verification of the MTL locus of the parental and mating product. (D) FACS analysis for the genomic DNA content of the representative mating product and parental strains. (E) Mating efficiency between the haploid and diploid strains on Lee’s GlcNAc at 25 °C for 7 days. The data underlying this figure can be found in S3 Data. The flow cytometry files are available from the Figshare database (https://doi.org/10.6084/m9.figshare.28350296). (TIF) [file pbio.3003062.s007.tif]

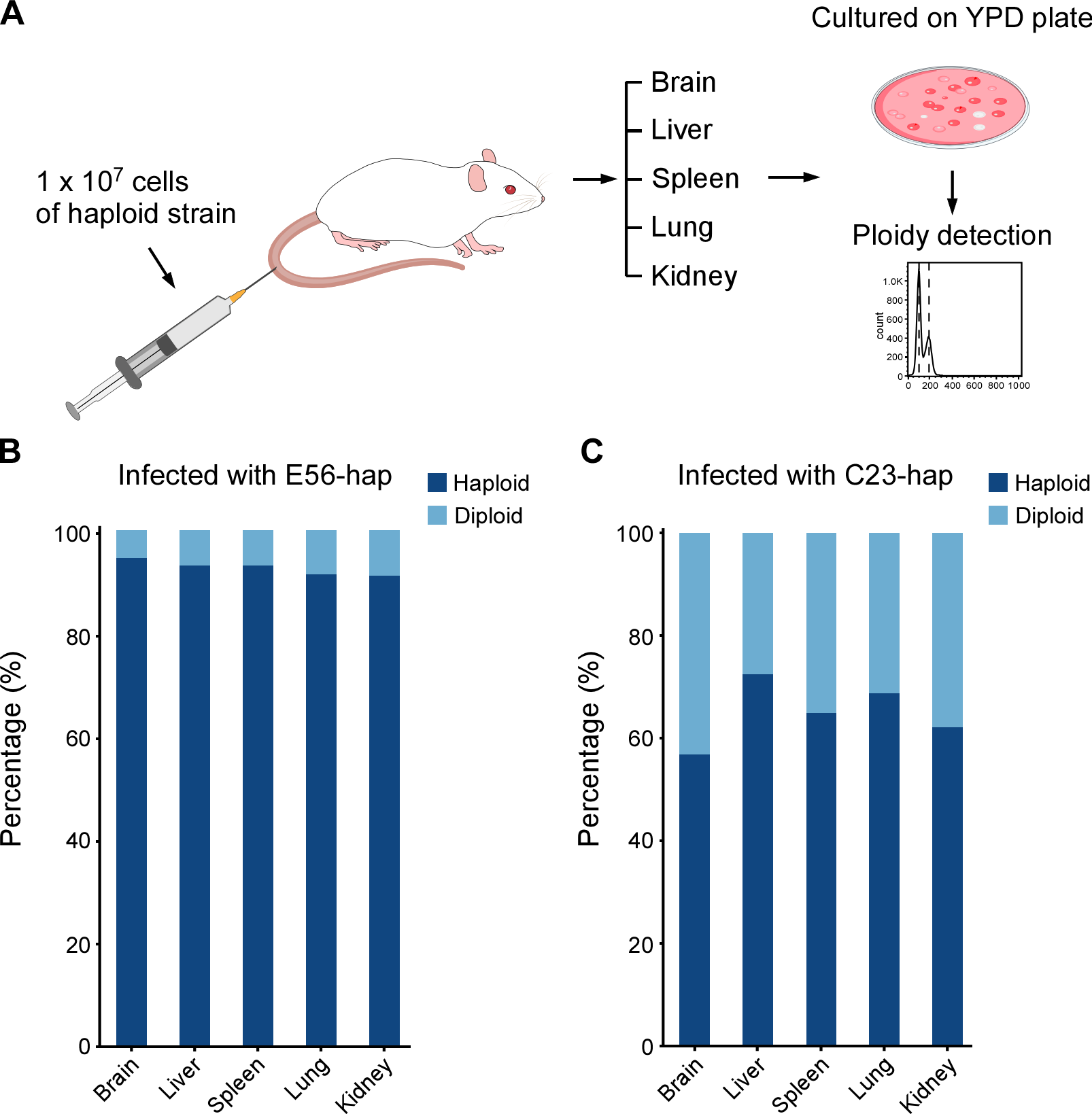

Supplement: S8 Fig — (A) Schematic of the ploidy stability test using the mouse infection model. (B) Percentages of the haploid and auto-diploidization colonies isolated from the mice systemically infected with strain E56-hap. (C) Percentages of the haploid and auto-diploidization colonies isolated from the mice systemically infected with strain C23-hap. Approximately 1 × 10^7^ haploid cells in 200 μL PBS were injected into mouse tail veins; fungal cells were recovered from the liver, kidney, brain, lung, and spleen 24 h post-infection, and replated onto YPD medium containing 5 μg/mL phloxine B. Ploidy states of all distinct colonies were inferred by FACS analysis. Bar plots represent the ploidy ratios of colonies isolated from the mice. The data underlying this figure can be found in S3 Data. (TIF) [file pbio.3003062.s008.tif]

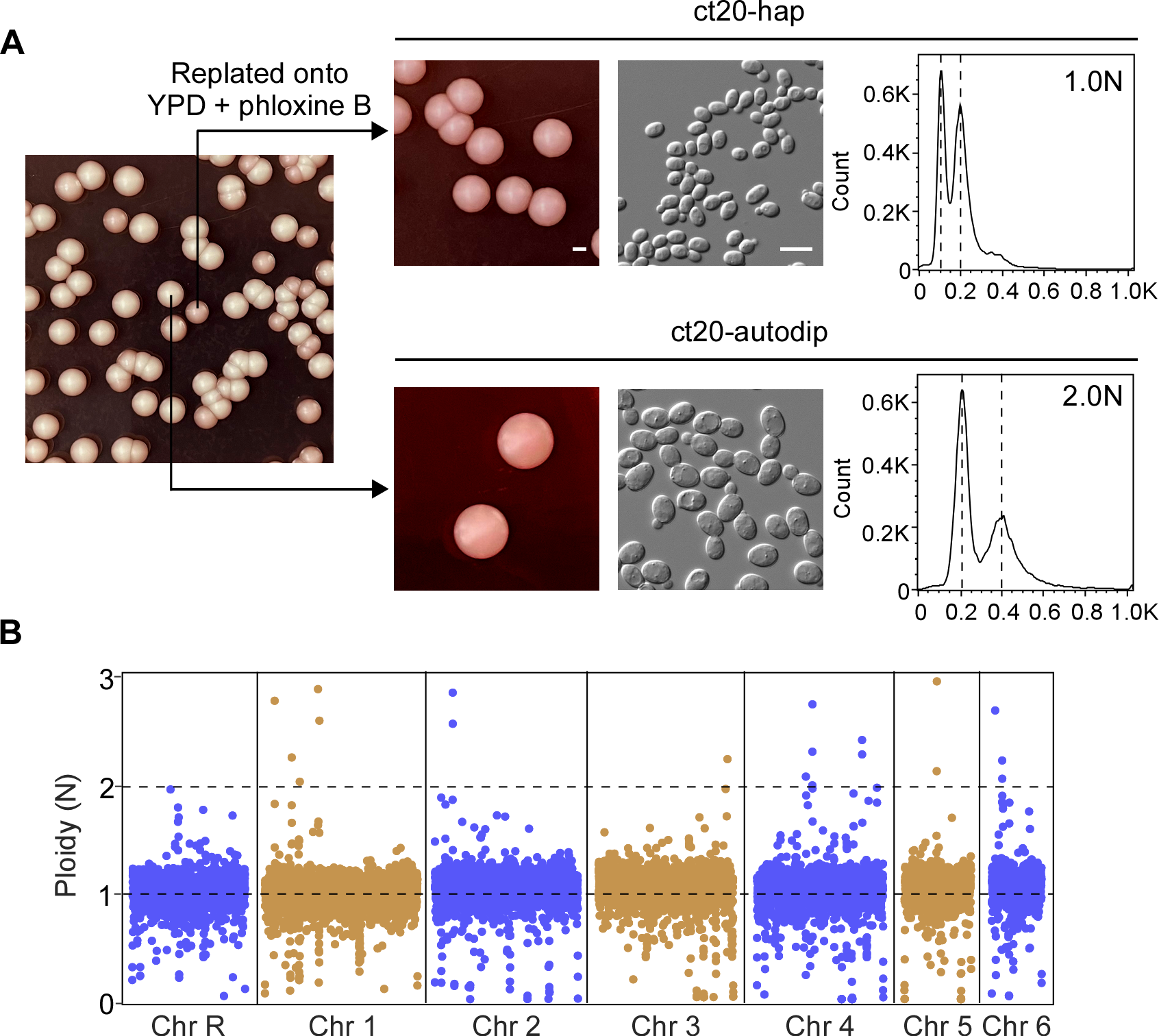

Supplement: S9 Fig — (A) Morphology of the natural haploid and auto-diploid strains. Fungal cells were plated onto YPD medium containing 5 µg/mL phloxine B and cultured at 30 °C for 3 days. Two distinct colonies were then replated onto a fresh YPD medium with phloxine B and cultured under the same condition. Scale bars for colonies and cells were 1 mm and 5 μm, respectively. (B) Scatter plot of the genome-wide copy number distribution. The x-axis represents the seven chromosomes and the y-axis represents the relative copy numbers. Each point indicates an average copy number of a genomic segment of 1,000 bp across the genome based on the coverage analysis of the genomic data. The flow cytometry files are available from the Figshare database (https://doi.org/10.6084/m9.figshare.28350296). (TIF) [file pbio.3003062.s009.tif]
