# Supplementary material for: An agricultural triazole induces genomic instability and haploid cell formation in the human fungal pathogen Candida tropicalis
Source: PLoS Biol. 2025 Apr 1;23(4):e3003062. doi: 10.1371/journal.pbio.3003062 (PMC11960876; doi:10.1371/journal.pbio.3003062)
Supplement: S1 Table — (DOCX) [file pbio.3003062.s013.docx]

**Table S1. Strains used in this study.**

| **Strain name** | **Parent strain** | **Genotype or description** | **Ploidy** | **Reference** |
| --- | --- | --- | --- | --- |
| GH1374 | - | Clinical isolate, *MTL***a**/**a** | 2.0N | [1] |
| E56 | - | Environmental isolate, *MTL***a**/ɑ | 2.0N | [2] |
| E57 | - | Environmental isolate, *MTL***a**/ɑ | 2.0N | [2] |
| E102 | - | Environmental isolate, *MTL***a**/ɑ | 2.0N | [2] |
| C23 | - | Clinical isolate, *MTL***a**/ɑ | 2.0N | [2] |
| C155 | - | Clinical isolate, *MTL***a**/ɑ | 2.0N | [2] |
| E56-a1 | E56 | Evolved isolate, *MTL***a**/ɑ | 2.3N | This study |
| E56-a2 | E56 | Evolved isolate, *MTL***a**/ɑ | 2.9N | This study |
| E56-a3 | E56 | Evolved isolate, *MTL***a**/ɑ | 2.7N | This study |
| E56-a4 | E56 | Evolved isolate, *MTL***a**/ɑ | 2.6N | This study |
| E56-a5 | E56 | Evolved isolate, *MTL***a**/ɑ | 2.5N | This study |
| E56-a6 | E56 | Evolved isolate, *MTL***a**/ɑ | 2.0N | This study |
| E56-hap | E56 | Evolved isolate, *MTL***a** | 1.0N | This study |
| E57-a1 | E57 | Evolved isolate, *MTL***a**/ɑ | 1.7N | This study |
| E57-a2 | E57 | Evolved isolate, *MTL***a**/ɑ | 1.9N | This study |
| E57-a3 | E57 | Evolved isolate, *MTL***a**/ɑ | 1.9N | This study |
| E57-a4 | E57 | Evolved isolate, *MTL***a**/ɑ | 1.9N | This study |
| E57-a5 | E57 | Evolved isolate, *MTL***a**/ɑ | 1.9N | This study |
| E57-a6 | E57 | Evolved isolate, *MTL***a**/ɑ | 2.5N | This study |
| E57-a7 | E57 | Evolved isolate, *MTL***a**/ɑ | 2.0N | This study |
| E102-a1 | E102 | Evolved isolate, *MTL***a**/ɑ | 2.6N | This study |
| E102-a2 | E102 | Evolved isolate, *MTL***a**/ɑ | 2.6N | This study |
| E102-a3 | E102 | Evolved isolate, *MTL***a**/ɑ | 2.6N | This study |
| E102-a4 | E102 | Evolved isolate, *MTL***a**/ɑ | 2.6N | This study |
| E102-a5 | E102 | Evolved isolate, *MTL***a**/ɑ | 2.2N | This study |
| E102-a6 | E102 | Evolved isolate, *MTL***a**/ɑ | 2.2N | This study |
| E102-a7 | E102 | Evolved isolate, *MTL***a**/ɑ | 2.0N | This study |
| C23-a1 | C23 | Evolved isolate, *MTL***a**/ɑ | 2.3N | This study |
| C23-a2 | C23 | Evolved isolate, *MTL***a**/ɑ | 2.2N | This study |
| C23-a3 | C23 | Evolved isolate, *MTL***a**/ɑ | 2.3N | This study |
| C23-a4 | C23 | Evolved isolate, *MTL***a**/ɑ | 3.1N | This study |
| C23-a5 | C23 | Evolved isolate, *MTL***a**/ɑ | 2.1N | This study |
| C23-a6 | C23 | Evolved isolate, *MTL***a**/ɑ | 2.0N | This study |
| C23-hap | C23 | Evolved isolate, *MTL*ɑ | 1.0N | This study |
| C155-a1 | C155 | Evolved isolate, *MTL***a**/ɑ | 2.3N | This study |
| C155-a2 | C155 | Evolved isolate, *MTL***a**/ɑ | 2.6N | This study |
| C155-a3 | C155 | Evolved isolate, *MTL***a**/ɑ | 2.5N | This study |
| C155-a4 | C155 | Evolved isolate, *MTL***a**/ɑ | 2.4N | This study |
| C155-a5 | C155 | Evolved isolate, *MTL***a**/ɑ | 2.9N | This study |
| C155-a6 | C155 | Evolved isolate, *MTL***a**/ɑ | 2.7N | This study |
| C155-a7 | C155 | Evolved isolate, *MTL***a**/ɑ | 2.0N | This study |

| **Strain name** | **Parent strain** | **Genotype or description** | **Ploidy** | **Reference** |
| --- | --- | --- | --- | --- |
| TH36 | E56-hap | *MTL***a**, *arg4-*, SAT1+ | 1.0N | This study |
| TH37 | C23-hap | *MTL*ɑ, *his1-*, SAT1+ | 1.0N | This study |
| CAY2060 | ATCC 34139 ST-120 | *arg4/arg4*, *MTL***a/a** | 2.0N | [3] |
| CAY2061 | ATCC 34139 ST-120 | *his1/his1*, *MTL*ɑ/ɑ | 2.0N | [3] |
| E56-autodip | E56-hap | *MTL***a**/**a** | 2.0N | This study |
| C23-autodip | C23-hap | *MTL*ɑ/ɑ | 2.0N | This study |
| ct20 | - | Clinical isolate | - | [4] |
| ct20-hap | ct20 | - | 1.0N | This study |
| ct20-autodip | ct20-hap | - | 2.0N | This study |

**Reference for Table S1:**

1. Zhang Q, Tao L, Guan G, Yue H, Liang W, Cao C, Dai Y, Huang G. Regulation of filamentation in the human fungal pathogen *Candida tropicalis*. Mol Microbiol. 2016, 99(3):528-45.
2. Hu T, Wang S, Bing J, et al. Hotspot mutations and genomic expansion of *ERG11* are major mechanisms of azole resistance in environmental and human commensal isolates of *Candida tropicalis*. Int J Antimicrob Agents. 2023, 62(6):107010.
3. Porman AM, Alby K, Hirakawa MP, Bennett RJ. Discovery of a phenotypic switch regulating sexual mating in the opportunistic fungal pathogen *Candida tropicalis*. Proc Natl Acad Sci U S A. 2011, 108(52):21158-63.
4. O'Brien CE, et al. Population genomics of the pathogenic yeast *Candida tropicalis* identifies hybrid isolates in environmental samples. PLoS Pathog. 2021, 17(3): e1009138.
