# Supplementary material for: An agricultural triazole induces genomic instability and haploid cell formation in the human fungal pathogen Candida tropicalis
Source: PLoS Biol. 2025 Apr 1;23(4):e3003062. doi: 10.1371/journal.pbio.3003062 (PMC11960876; doi:10.1371/journal.pbio.3003062)
Supplement: S2 Table — (DOCX) [file pbio.3003062.s014.docx]

| **No.** | **Name** | **Sequence (5' to 3')** | **Purpose** |
| --- | --- | --- | --- |
| 1 | CtA1-F101 | AGGCAGATGATTTTCGAGCC | For verification of *MTL***a**1 in *C. tropicalis* |
| 2 | CtA1-R424 | ATTCCTTCTTGGAAGAATCCGGT |  |
| 3 | CtAlpha2-F12 | CTGGGATACCCTTCACCAAGTC | For verification of *MTL*α2 in *C. tropicalis* |
| 4 | CtAlpha2-R400 | TCTGGAACCACCCTTCCAAT |  |
| 5 | CtHis1-up-5’ (his1) | CTATATGGGCCCAGACACGAT  CAATACATCTG | Construction of plasmid pSFS2A-HIS1-KO |
| 6 | CtHis1-up-3’ (his1) | TGGTTACTCGAGACGGCAAAT  AATAAACGGTC |  |
| 7 | CtHis1-down-5’ (his1) | TTTGGCCCGCGGTGTAGAGTT  TAGAGTTCATC |  |
| 8 | CtHis1-down-3’ (his1) | TTCACTGAGCTCACATTCAATT  GTACTCGATG |  |
| 9 | CtHis1-5’ check (his1) | TAGGTACATGGACGAAATGG | *HIS1* deletion confirmation |
| 10 | CtHis1-3’ check (his1) | AATAGCATACACTGGTGAGG |  |
| 11 | 5-detect (pSFS2a) | CAATGAAATCCAGACAGTC GAG |  |
| 12 | 3-detect (pSFS2a) | CGATTAGAGACACAAACGA AC |  |
| 13 | CtHis1-ORF-5’ (his1) | AGTCCAAGTTCCAGCTAATG |  |
| 14 | CtHis1-ORF-3’ (his1) | AGTAGAGACAGTGGCAGCTC |  |
| 15 | CtArg4-up-5’ (his1) | TTATATGGGCCCTTACTGG  AGCTACTGATCCAC | Construction of plasmid pSFS2A-ARG4-KO |
| 16 | CtArg4-up-3’ (his1) | AATCATCTCGAGTACCAGT  ATGAACTTTACCAG |  |
| 17 | CtArg4-down-5’ (his1) | TAATATCCGCGGGCTGATGC  TTATTCAACCGG |  |
| 18 | CtArg4-down-3’ (his1) | ATATACGAGCTCGTAGCCAAC  ATGTCCATAGT |  |
| 19 | CtArg4-5’ check (arg4) | TCGTCGACTTTTCTTTCTTTCT | *ARG4* deletion confirmation |
| 20 | CtArg4-3’ check (arg4) | CACTTAATCCTTCTTCTTCAGC |  |
| 21 | CtArg4-ORF-5’ (arg4) | TCATTTACAACGTGCCCAAC |  |
| 22 | CtArg4-ORF-3’ (arg4) | CTGCACTTGAATAAAGAATC |  |

**Table S2. Primers used in this study.**

| **No.** | **Name** | **Sequence (5' to 3')** | **Purpose** |
| --- | --- | --- | --- |
| 23 | ACT1-F | TTTACGCTGGTTTCTCCTTGC | q-RT-PCR of *ACT1* |
| 24 | ACT1-R | GCAGCTTCCAAACCTAAATCG |  |
| 25 | ERG11-F | TGCCTGGTTCTTGTTGCATTT | q-RT-PCR of *ERG11* |
| 26 | ERG11-F | AATCGTTCAAGTCACCACCCT |  |
| 27 | TAC1-F | CAGGTAAACCGCCAATGATT | q-RT-PCR of *TAC1* |
| 28 | TAC1-R | GACGATTGTTCTGTGGTTGATC |  |
| 29 | ERG5-F | AGATTCTGTAAGTACGACGG | q-RT-PCR of *ERG5* |
| 30 | ERG5-R | TGGTATCATCAGCAATCTTCT |  |
| 31 | ERG3-F | TGGAAATCGGTTTGGCAACT | q-RT-PCR of *ERG3* |
| 32 | ERG3-R | AGGAAATTGCCATAAAAGTGCCT |  |
| 33 | ERG26-F | TTTATACTTCCTCAGCTGGTG | q-RT-PCR of *ERG26* |
| 34 | ERG26-R | GGAACTAATTGACGATCTCCT |  |
| 35 | HMG1-F | GTAAGGCTGTTTCCTCCCAAT | q-RT-PCR of *HMG1* |
| 36 | HMG1-R | CCAAAACGTATTGCAAATCTT |  |
| 37 | RCH1-F | TTGGAGCATTTATAACCCCTG | q-RT-PCR of *RCH1* |
| 38 | RCH1-R | TGACTAACACTCGTAAAAGCA |  |
| 39 | ROA1-F | GCTATCACAATTGGATGGATG | q-RT-PCR of *ROA1* |
| 40 | ROA1-R | AGTTTGTAATCCCCACATGAA |  |
| 41 | CDR1-F | GAGGTTTGGATTCCGCTAC | q-RT-PCR of *CDR1* |
| 42 | CDR1-R | GGCTTTGTCTGCTTTCCCA |  |
| 43 | MDR1-F | GTGCATCATTCCAGCCTA | q-RT-PCR of *MDR1* |
| 44 | MDR1-R | GGATGGCAATCATCACGAG |  |
